# Catheter ablation for atrial fibrillation and impact on clinical outcomes

**DOI:** 10.1093/ehjopen/oeae058

**Published:** 2024-07-15

**Authors:** Rui Providencia, Hussam Ali, Antonio Creta, Sérgio Barra, Prapa Kanagaratnam, Richard J Schilling, Michal Farkowski, Riccardo Cappato

**Affiliations:** Institute of Health Informatics Research, University College London, 222 Euston Road, London NW1 2DA, UK; Barts Heart Centre, St Bartholomew's Hospital, Barts Health NHST Trust, W Smithfield, London EC1A 7BE, UK; Arrhythmia and Clinical Electrophysiology Center, IRCCS, MultiMedica, Via Milanese, 300, 20099 Sesto San Giovanni, Milan, Italy; Institute of Health Informatics Research, University College London, 222 Euston Road, London NW1 2DA, UK; Barts Heart Centre, St Bartholomew's Hospital, Barts Health NHST Trust, W Smithfield, London EC1A 7BE, UK; Department of Cardiology, Hospital da Luz Arrábida, Praceta de Henrique Moreira 150, 4400-346 Vila Nova de Gaia, Portugal; National Heart and Lung Institute, Hammersmith Campus, Imperial College London, 72 Du Cane Road, W12 0HS London, UK; Department of Cardiology, Hammersmith Hospital, Imperial College Healthcare NHS Trust, 72 Du Cane Road, W12 0HS London, UK; Barts Heart Centre, St Bartholomew's Hospital, Barts Health NHST Trust, W Smithfield, London EC1A 7BE, UK; William Harvey Research Institute, Faculty of Medicine and Dentistry, Queen Mary University of London, Charterhouse Square London, EC1M 6BQ London, UK; Department of Cardiology, Ministry of Interior and Administration National Medical Institute, ul. Wołoska 137, 02-507 Warszawa, Poland; Arrhythmia and Clinical Electrophysiology Center, IRCCS, MultiMedica, Via Milanese, 300, 20099 Sesto San Giovanni, Milan, Italy

**Keywords:** Arrhythmia, Percutaneous intervention, Rhythm control, Paroxysmal, Persistent and stroke

## Abstract

**Aims:**

Catheter ablation is the most effective rhythm-control option in patients with atrial fibrillation (AF) and is currently considered an option mainly for improving symptoms. We aimed to assess the impact of catheter ablation on hard clinical outcomes.

**Methods and results:**

We performed a systematic review of randomized controlled trials (RCTs) comparing catheter ablation vs. optimized medical treatment. We searched MEDLINE, EMBASE, and CENTRAL on 8 January 2024, for trials published ≤10 years. We pooled data through risk ratio (RR) and mean differences (MDs), with 95% confidence interval (CI), and calculated the number needed to treat (NNT). Sub-group and sensitivity analyses were performed for the presence/absence of heart failure (HF), paroxysmal/persistent AF, early ablation, higher/lower quality, and published ≤5 vs. >5 years. Twenty-two RCTs were identified, including 6400 patients followed for 6–52 months. All primary endpoints were significantly reduced by catheter ablation vs. medical management: all-cause hospitalization (RR = 0.57, 95% CI 0.39–0.85, *P* = 0.006), AF relapse (RR = 0.48, 95% CI 0.39–0.58, *P* < 0.00001), and all-cause mortality (RR = 0.69, 95% CI 0.56–0.86, *P* = 0.0007, NNT = 44.7, driven by trials with HF patients). A benefit was also demonstrated for all secondary endpoints: cardiovascular mortality (RR = 0.55, 95% CI 0.34–0.87), cardiovascular (RR = 0.83, 95% CI 0.71–0.96), and HF hospitalizations (RR = 0.71, 95% CI 0.56–0.89), AF burden (MD = 20.6%, 95% CI 5.6–35.5), left ventricular ejection fraction (LVEF) recovery (MD = 5.7%, 95% CI 3.5–7.9), and quality of life (MLHFQ, AFEQT, and SF-36 scales).

**Conclusion:**

Catheter ablation significantly reduced hospitalizations, AF burden, and relapse, and improved quality of life. An impact on hard clinical outcomes, with an important mortality reduction and improvement in LVEF, was seen for patients with AF and HF.

## Introduction

Atrial fibrillation (AF) is the most frequent sustained arrhythmia^1^ and has a great impact on healthcare utilization.^2^ It affects 52.55 million individuals globally (0.67% of worldwide population), and is a growing health problem, having increased by 86% over the last 30 years.^3^

Atrial fibrillation causes symptoms (e.g. palpitations, shortness of breath, fatigue, chest pain) and is associated with cardiovascular complications [e.g. ischaemic stroke, heart failure (HF), vascular dementia], as well as increased all-cause mortality.^4^

Management of AF relies on anticoagulants for prevention of stroke and systemic embolism in patients at risk,^5,6^ attempting to restore and preserve normal sinus rhythm (rhythm control—using anti-arrhythmic drugs (AAD), or a procedure called catheter ablation) or controlling the heart rate to prevent tachycardia-related symptoms (rate control), and controlling/treating associated risk factors and comorbidities.

Catheter ablation’s current main indication is for the management of symptoms in AF patients where AAD have proven to be ineffective.^7,8^ Whilst in the early 2000s rhythm control was not thought to have any particular benefit on hard clinical outcomes,^9,10^ more recent data suggest potential clinical benefits of this strategy,^11^ namely through the use of catheter ablation.^12,13^ In addition, since its development in 1998,^14^ AF ablation technology and operator experience have evolved, and procedural complications and success rates have improved over time.^15^

We aimed to assess the potential clinical benefit of catheter ablation using data from contemporary randomized controlled trials (RCTs; i.e. published over the past 10 years), to provide a potential evidence support for guideline recommendations for this procedure.

## Methods

We undertook a systematic review according to the Preferred Reporting Items for Systematic Reviews and Meta-analysis (PRISMA) Statement and recommendations stated in the Cochrane Handbook for Systematic Reviews of Interventions.^16^ In our review, we included exclusively RCTs at the patient level reported as full-text or published as abstract only.

The registration date for PROSPERO is 15 November 2023. PROSPERO 2023 CRD42023483166 available from: https://www.crd.york.ac.uk/prospero/display_record.php? ID=CRD42023483166.

### PICO framework

We followed the PICO framework (P—patient, I—intervention, C—comparison, O—outcome) to guide our systematic review. Participants were all patients aged ≥ 18 years with AF of any type and duration. The intervention of interest was catheter ablation for AF consisting of pulmonary vein isolation ± additional lesion sets (i.e. lines, complex fractionated atrial electrogram ablation, rotors, areas of focal activity). Standard medical therapy, which may include rate control or rhythm control agents, was the comparator. The primary outcomes for this review were: All-cause mortality; HF hospitalizations; and AF relapse.

Cardiovascular mortality; Hospitalizations; Cardiovascular Hospitalizations; Change in left ventricular ejection fraction (LVEF); Change in Quality of Life (measured using the Minnesota Living with Heart Failure Questionnaire—MLHFQ, Atrial Fibrillation Effect on QualiTy-of-life—AFEQT, and the 36-Item Short Form Health Survey—SF-36); and change in AF burden measured continuously via implantable or wearable cardiac monitor, or implantable cardiac device were the secondary outcomes.

### Search strategy

We performed a search on MEDLINE, EMBASE, and Cochrane Central Register of Controlled Trials (CENTRAL) from the 1^st^ January 2014 to the 8^th^ January 2024 for identifying potentially eligible RCTs for our systematic review. Details of the search are provided in Supplementary material online, Annex A.

We included all RCTs published over the past 10 years (date of first publication: 1 January 2014 to 8 January 2024) with the abovementioned population, intervention and comparison, and providing data on stroke. Two authors (R.P. and H.A.) screened the search results independently. In cases of disagreement, a third author (A.C.) was involved for the final decision.

Recently published systematic reviews on the same topic were revised to confirm no important trials had been missed on the initial search strategy.

### Data extraction

We included all RCTs with the abovementioned population, intervention, and comparison, and providing data for at least one outcome.

Data on study design, population, country, outcomes, baseline population variables [% of paroxysmal, persistent AF, age, sex, CHA_2_DS_2_VASc score, HF, diabetes mellitus, hypertension, valvular heart disease, hypertrophic cardiomyopathy (HCM)], ablation strategy, settings and technology, AAD and other cardiovascular drugs used in the control group, follow-up duration, and method of follow-up (i.e. Holter, implantable cardiac monitor, etc.) were extracted and checked by two review authors (S.B., H.A., or A.C.), who resolved any differences by discussion and consensus. When agreement was not reached, a third review author (R.P.) was contacted for a final decision. When necessary, we contacted the authors of primary studies for additional information.

### Quality appraisal

We applied the Cochrane ‘Risk of bias’ tool version 1 by assessing the following domains: Random sequence generation, allocation concealment, blinding of participants and personnel, blinding of outcome assessment, incomplete outcome data, selective outcome reporting, and other bias (e.g. evidence of prospective trial registration). These were assessed and judged as high, low, or unclear risk of bias by two review authors (M.F. and R.P.) who independently assessed the risk of bias for each included study. Disagreements were resolved by consultation with a third review author (A.C.) or by general consensus.

Taking into account that included trials are assessing a procedure vs. best medical therapy, we acknowledged there will be issues with blinding of patients and personnel. Hence, for objective outcomes like ‘all-cause mortality’, ‘stroke’, and ‘AF Burden’, we considered this domain to be ‘low risk’ as it is highly unlikely that knowledge of the assigned treatment could influence one of these outcomes.

We used GRADE system to assess the quality of the body of evidence associated with the selected outcomes in our review.^17^ The GRADE approach appraises the quality of a body of evidence based on the extent to which one can be confident that an estimate of effect or association reflects the item being assessed. The quality measure of a body of evidence considers within-study risk of bias, the directness of the evidence, heterogeneity of the data, precision of effect estimates, and risk of publication bias.

The decision to downgrade the certainty of evidence resulted from a consensus between two review authors (R.P. and A.C.), and whenever needed a third review author (M.F.) intervened. We added a footnote to the ‘Summary of findings’ table to explain which domains were taken into account in the decision.

#### Sub-group and sensitivity analyses

We performed sub-group and sensitivity analyses for studies with:

– Heart failure populations vs. no HF or mixed populations (normal population with small percentage of HF patients).
– Studies of paroxysmal AF vs. persistent AF vs. mixed populations (paroxysmal + persistent AF).
– Early ablation (i.e. ablation as first-line therapy) vs. Ablation after drug failure.
– Studies published over the last 5 years (2019–23) vs. the previous 5-year period (2014–18).
– Higher quality studies (up to 1 domain with high risk of bias) vs. Lower quality studies (studies with two or more domains with high risk of bias).

#### Strategy for data synthesis

We performed a meta-analysis for all outcomes utilizing risk ratio (RR) for rates, with a 95% confidence interval (CI), or mean difference (MD) (± standard deviation). Standardized MD was used when required (i.e. when different scales are used for reporting) for continuous data.

The number needed to treat (NNT) or number needed to harm (NNH) were calculated,^18^ whenever applicable, for the pre-specified review endpoints. This was estimated as the reciprocal of the absolute risk difference for the particular outcome between treated subjects and the control or placebo group—that is


$$\mathrm{NNT}=\;\frac{1}{\mathrm{Absolute}\;\mathrm{Ris}k_{\mathrm{Control}\;\mathrm{Group}}-\mathrm{Absolute}\;\mathrm{Ris}k_{\mathrm{Treatment}\;\mathrm{Group}}}$$

Heterogeneity was assessed using the *I*² statistic. We followed the recommendations for thresholds in the Cochrane Handbook for Systematic Reviews of Interventions:^19^ 0–40%: might not be important; 30–60%: may represent moderate heterogeneity; 50–90%: may represent substantial heterogeneity; and 75–100%: may represent considerable heterogeneity.

We assessed publication bias and other reporting biases by visual inspection of funnel plots for primary outcomes when at least 10 trials were included.^19^ Using visual assessment of the asymmetry of the funnel plot we will assess the risk of reporting bias.

Random or fixed effects models were used, as appropriate. Review Manager (RevMan 5.3) was utilized to perform all the necessary analyses.

## Results

### Search results and included trials

**Figure 1:**
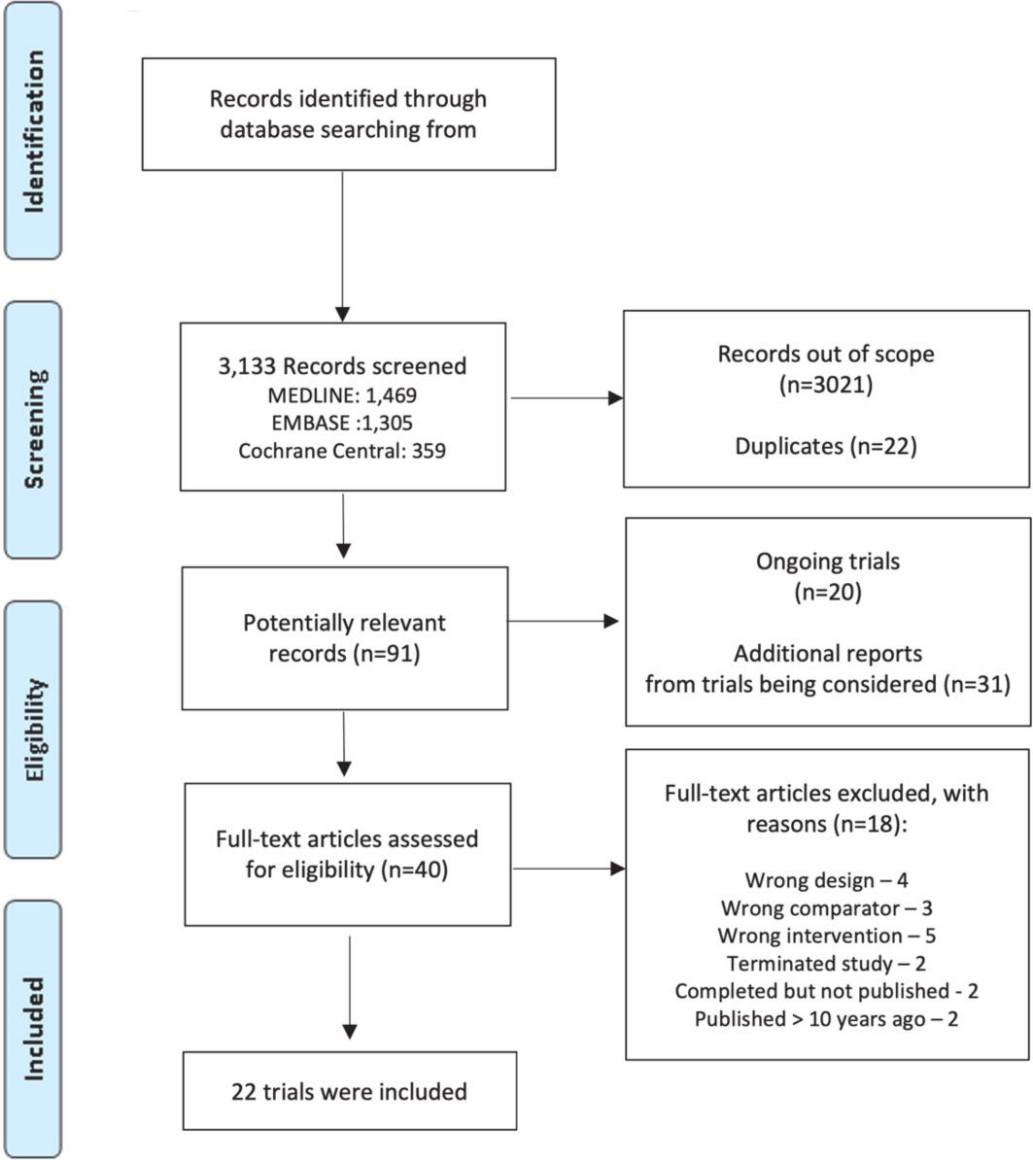
PRISMA flowchart with study selection.

Our searches on MEDLINE, EMBASE, and Cochrane CENTRAL identified 3133 records (*Figure 1*). After excluding duplicates, and records based on analysis of the abstract, we identified 40 records whose full manuscript was assessed for eligibility. In the end, this search yielded 22 trials that met the inclusion criteria for our review.^12,13,16,20–38^ A list of excluded studies and reasons is presented in Supplementary material online, *Table S1*). Twenty-nine additional reports (protocol, sub-studies, etc.) referring to the included trials were identified and were utilized when required to inform this review (see Supplementary material online, *Table S2*). With the aim of investigating the ongoing research in this field, and selecting potential studies for future updates, we identified 22 ongoing trials of potential interest (see Supplementary material online, *Table S3*). We also carefully screened nine recently published systematic reviews on catheter ablation of AF vs. medical therapy^39–47^ and could not identify any further trials meeting criteria for our systematic review.

The 22 eligible RCTs, included a total of 6400 unique patients followed for a range of 6–52 months. Mean age across trials was in the 50 s or 60 s, and women were less represented (rate varied from 3.8% to 50%). All included patients had no previous catheter ablation procedures for AF. At the end of follow-up, the mean number of ablation procedures varied across studies, ranging from single ablation,^22,27,31,36,38^ to 1.7 ablations per patient^28^ or repeat ablation procedure in 45.0%^26^ (see Supplementary material online, *Table S4*).

Five trials included paroxysmal AF patients only,^16,23,24,27,31^ two trials included >95%,^25,29^ eight included persistent AF patients only,^26,28,30,32,33,35,37,38^ one trial included >90% patients with persistent AF^21^ and the remaining included a mix of paroxysmal and persistent AF.^12,13,20,22,34,36^ Most trials were conducted in North America and Europe, and 16 were published in 2019 or afterwards. *Table 1* provides information on the included trials baselines and study design.

**Table 1:** Included trials—study design and baselines

Study centres	Country	*n*	Paroxysmal AF	Age	Women	*n* of failed AADs	BMI (Kg/m^2^)	Valvular/HCM	CHA_2_DS_2_VASc	HF	LVEF %
CAMTAF 2014 Single-centre	UK	Abl 26 Med 24	0% (0)	Abl 55 ± 12 Med 60 ± 10	Abl 3.8% (1) Med 12.5% (3)	Abl 1 (IQR 0–1) Med 1 (IQR 0–1)	NA	NA	NA	100% (50)	<50%
RAAFT-2 2014 Multicentre	Canada, USA, and Germany	Abl 66 Med 61	Abl 99% (65) Med 97% (59)	Abl 56 ± 9 Med 54 ± 12	Abl 23% (15) Med 26% (16)	Abl 0 Med 0	NA	0% (0)	CHADS_2_ Abl 0 (IQR 0–1) Med 0 (IQR 0–1)	Abl 3% (2) Med 2% (1)	>40%
AATAC 2016 Multicentre	USA, Italy, France, and Czechia	Abl 102 Med 101	0% (0)	Abl 62 ± 10 Med 60 ± 11	Abl 25% (27) Med 27% (28)	NA	Abl 30 ± 8 Med 29 ± 4	NA	NA	100% (203)	≤40%
Sohara et al. 2016 Multicentre	Japan	Abl 100 Med 43	100%	Abl 59 ± 10 Med 61 ± 10	Abl 20% (20) Med 19% (8)	Refractory to ≥1 Class I–IV AAD	NA	NA	CHADS_2_ Abl 1 ± 1 Med 1 ± 1	NA	Abl 67 ± 6 Med 67 ± 7
CAMERA-MRI 2017 Multicentre	Australia	Abl 33 Med 33	0% (0)	Abl 59 ± 11 Med 62 ± 9.4	Abl 6% (2) Med 12% (4)	Previous use of Amiodarone in 82–91%	Abl 30 ± 7 Med 31 ± 4	NA	CHA_2_DS_2_VASc 2.4 ± 1.0	100% (66)	Abl 32 ± 9 Med 34 ± 8
CASTLE-AF 2018 Multicentre	USA, Germany, Netherlands, Hungary, and Russia	Abl 179 Med 184	Abl 30% (54) Med 35% (64)	Abl 64 (IQR 56–71) Med 64 (IQR 56–74)	Abl 13% (23) Med 16% (29)	Previous use of Amiodarone in 57–60%	Abl 29 (26–32) Med 29 (26–32)	NA	NA	100% (363)	≤35%
AMICA 2019 Multicentre	Germany and Hungary	Abl 68 Med 72	0% (0)	Abl 65 ± 8 Med 65 ± 8	Abl 12% (8) Med 8% (6)	NA	NA	Valvular Abl 2% (2) Med 1% (1)	NA	100% (140)	≤35%
CABANA 2019 Multicentre	USA, Australia, Canada, China, Czechia, Germany, Italy, Korea, Russia, and UK	Abl 1108 Med 1096	Abl 42.4% (470) Med 43.5% (476)	Abl 68 (IQR 62–72) Med 67 (IQR 62–72)	Abl 37.3% (413) Med 37.0% (406)	Previous use of 1 AAD in 82.2% ≥ 2 AAD in 17.8%	Abl 30 (IQR 27–34) Med 30 (IQR 26–35)	NA	3 (IQR 2–4)	Abl 34.1% (378) Med 36.5% (400)	≤35% Abl 4.8% (38) Med 4.2% (31)
CAPAPAF 2019 Single-centre	UK	Abl 23 Med 22	0% (0)	Abl 75 Med 72	Abl 52% (12) Med 45% (10)	NA	Abl 29 ± 8 Med 29 ± 6	Valvular Abl 22%(5) Med 14% (3)	Mean: Abl 4 Med 3	NA	NA
CAPTAF 2019 Multicenter	Sweden and Finland	Abl 79 Med 76	70.9% (56) 75% (57)	Abl 55.8 ± 10.6 Med 56.3 ± 8.9	Abl 26.6% (21) Med 18.4% (14)	Previous use of AAD in Abl 38.0% (30) Med 44.7% (34)	Abl 27 ± 4 Med 27 ± 4	Valvular 1.3% (1)	Abl 1 (IQR 0–2) Med 1 (IQR 0–1)	Abl 2.5% (2) Med 3.9% (3)	Abl 56 ± 7 Med 56 ± 8
ATTEST 2020 Multicentre	Germany, Russia, Hungary, Latvia, Korea, and Italy	Abl 128 Med 127	100%	Abl 67.8 ± 4.8 Med 67.6 ± 4.6	Abl 57.8% (74) Med 58.2% (74)	Previous use of AAD in Abl 47.7% (61) Med 54.3% (69)	NA	0% HCM	NA	Abl 18.8% (24) Med 21.3% (21)	Abl 62 ± 6 Med 62 ± 5
STOP AF First 2020 Multicentre	USA	Abl 104 Med 99	100%	Abl 60.4 ± 11.2 Med 61.16 ± 11.2	Abl 39.4% (41) Med 42.4% (42)	0%	NA	Valvular Abl 8% (8) Med 9% (9)	CHA_2_DS_2_-VASc ≥3: Abl 22% (23) Med 36.3% (36)	Abl 1% (1) Med 3% (3)	Abl 61 ± 6 Med 61 ± 6
EARLY-AF 2021 Multicentre	Canada	Abl 154 Med 149	Abl 95.5% (147) Med 94.0% (140)	Abl 57.5 ± 12.3 Med 59.5 ± 10.6	Abl 27.2% (42) Med 31.5% (47)	Remote/irregular or under-dosed: Abl 26% (40) Med 29.5% (44)	Abl 31 ± 14 Med 30 ± 9	NA/Excluded *LVH >18 mm, >moderate-severe MR, prothesic valves*	Abl 1.9 ± 1.0 Med 1.9 ± 1.1	Abl 9.1% (14) Med 9.4% (14)	Abl 60 ± 7 Med 60 ± 8
CAPA 2021 Multicentre	China	Abl 327 Med 321	0%	Abl 64.8 ± 12.6 Med 64.4 ± 13.6	Abl 33.3% (109) Med 36.7% (118)	Previous use of Amiodarone in Abl 73.7% (241) Med 68.8% (221)	NA	NA/excluded HCM	Abl 2.0 ± 0.9 Med 2.1 ± 1.0	0%	Abl 53 ± 9 Med 52 ± 9
Cryo-FIRST 2021 Multicentre	Germany, Italy, Belgium, Croatia, France, Norway, and USA	Abl 107 Med 111	100%	Abl 50.5 ± 13.1 Med 54.1 ± 13.4	Abl 28.9% (31) Med 35.1% (39)	0%	NA	Valvular Abl 2.8% (3) Med 1.8% (2) HCM 0%	CHA_2_DS_2_VASc ≥ 3: Abl 6.5% (7) Med 10.8% (12)	0%	Abl 63 ± 5 Med 64 ± 5
RAFT-AF 2022 Multicentre	Canada, Brazil, Sweden and Taiwan	Abl 214 Med 197	Abl 8.9% (19) Med 5.6% (11)	Abl 65.9 ± 8.6 Med 67.5 ± 8.0	Abl 26.6% (57) Med 24.9% (49)	Previous use of AAD in Abl 43.9% (94) Med 39.1% (77)	Abl 30 ± 7 Med 31 ± 7	NA Excluded severe valvular or rheumatic	CHA_2_DS_2_VASc ≥ 3: Abl 65.8% (141) Med 70.5% (139)	100%	≤45% in Abl 57.9% (124) Med 58.9% (116)
AVATAR 2022 Multicentre	UK	Abl 218 Med 103	100%	Abl 59.9 ± 10.6 Med 60.5 ± 10.3	Abl 40.4% (88) Med 44.6% (46)	Previous use of AAD in 1/3	Abl 29 ± 5 Med 28 ± 5	NA	CHA_2_DS_2_VASc ≥ 3: Abl 16.0% (35) Med 22.5% (23)	NA	Abl 58 ± 5 Med 58 ± 6
Ding et al. 2022 Single-centre	China	Abl 102 Abl 102	0% (0)	Abl 60.9 ± 7.9 Med 60.7 ± 10.2	Abl 40.2% (41) Med 41.2% (42)	0% Amiodarone	Abl 26 ± 3 Med 26 ± 3	NA	Abl 1.7 ± 1.4 Med 1.8 ± 1.3	Abl 6.9% (7) Med 9.8% (10)	Abl 61 ± 5 Med 60 ± 5
CASTLE-HTx 2023 Single-centre	Germany	Abl 97 Med 97	Abl 29% (28) Med 32% (31)	Abl 62 ± 12 Med 65 ± 10	Abl 12.3% (12) Med 25.7% (25)	Previous use of Amiodarone in Abl 45% (44) Med 47% (46)	Abl 28 ± 4 Med 28 ± 5	NA	NA	100% (endstage pre-transplant)	Abl 29 ± 6 Med 25 ± 6
RCT-STALL HFpEF 2023 Single-centre	Australia	Abl 16 Med 15	Abl 18.8% (3) Med 20% (3)	Abl 65.5 ± 7.6 Med 66.7 ± 7.9	Abl 50% (8) Med 53.3% (8)	Previous use of AAD in Abl 68.75% (11) Med 66.66% (10)	Abl 31 ± 6 Med 32 ± 4	0%/excluded	CHA_2_DS_2_VASc Abl 3 ± 2 Med 4 ± 2	100% (HFpEF)	Abl 60 ± 5 Med 59 ± 5
REMEDIAL 2023 Multicentre	Australia	Abl 49 Med 47	Abl 47% (23) Med 62%(29)	Abl 58 ± 13 Med 60 ± 11	Abl 35% (17) Med 30% (14)	NA	Abl 30 ± 5.5 Med 31 ± 7	NA	Median Abl 1.5 (IQR 1–3) Med 1 (IQR 0–2)	Abl 12% (6) Med 4% (2)	Abl 57 ± 8 Med 61 ± 5
ORBITA-AF feasibility 2024 Single-centre	UK	Abl 10 Med 10	0% (0)	Abl 69 ± 6 Med 72 ± 7	Abl 30% (3) Med 30% (3)	Previous use of 1–3 AADs, including calcium channel blockers, Beta-blockers and Amiodarone	Abl 26 ± 5 Med 28 ± 4	NA	NA	NA	Abl 44 ± 10 Med 52 ± 11

IQR, interquartile range.

Utilization of AAD prior to catheter ablation varied, with four trials referring patients for an early ablation strategy.^23,25,27,29^ Use of anticoagulants was comparable and as per guideline-recommendations^7,8^ Eight studies included only patients with HF,^12,13,21,22,28,30,32,33^ most with reduced LVEF,^12,13,21,28,30,32,33^ and one trial with preserved LVEF.^22^ Sohns et al.^13^ included patients on the waiting list for heart transplant. Information on post-procedure management, monitoring, and complications is provided in Supplementary material online, *Tables S4* and *S5*.

### Main analyses

**Figure 2:**
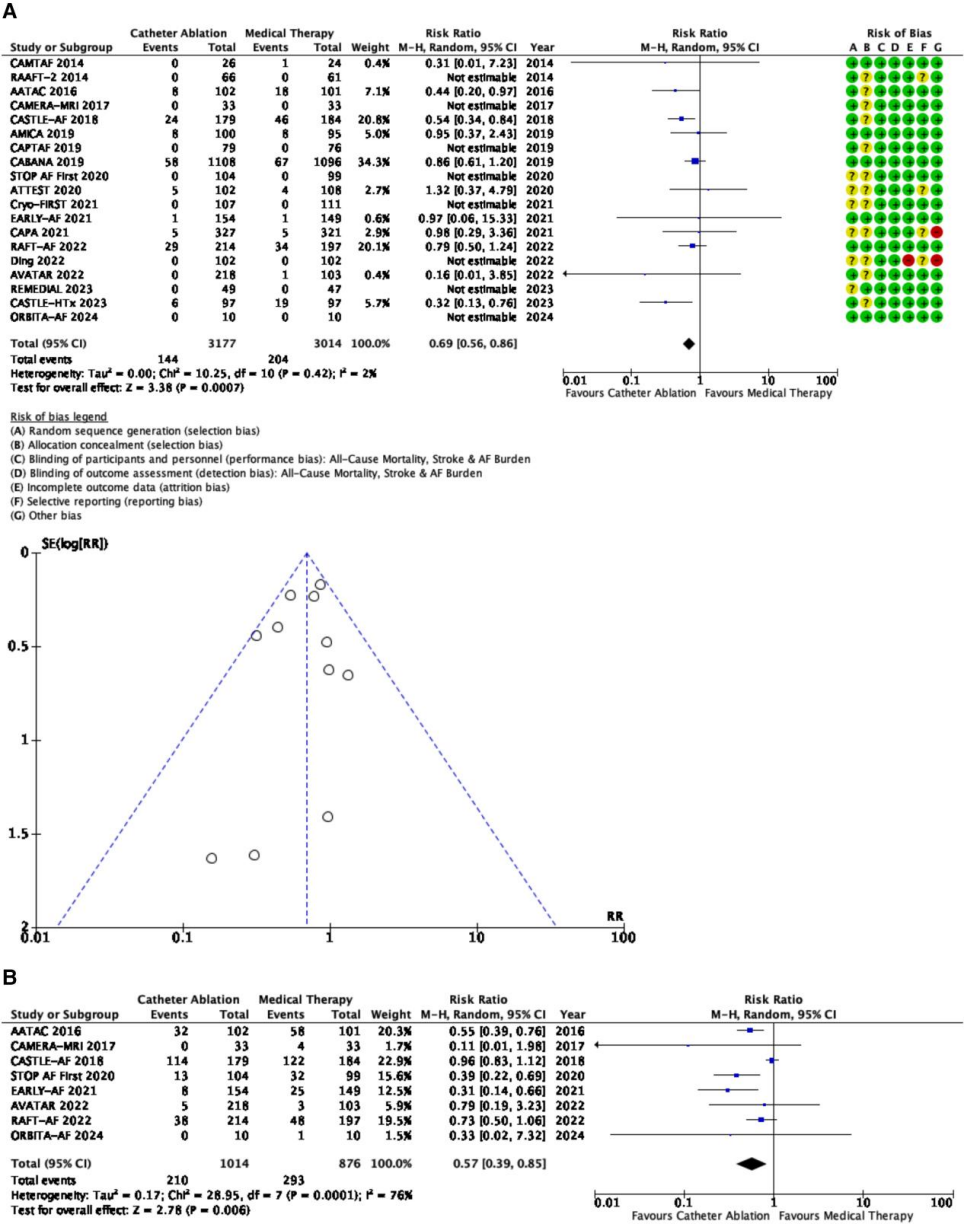
(*A*) Analyses for all-cause mortality and (*B*) all-cause hospitalizations.

*Figure 2A* illustrates in detail the positive impact of catheter ablation on all-cause mortality (RR: 0.69, 95% CI 0.56–0.86, *P* = 0.0007, *I*^2^ = 2%, with 3 out of 16 studies showing a significant benefit, NNT = 44.7), with no publication bias was identified by the funnel plots. Catheter ablation was associated with a significant reduction in all-cause hospitalizations (RR: 0.57, 95% CI 0.39–0.85, *P* = 0.006, *I*^2^ = 75%, with 3 out of 8 studies showing a significant benefit, NNT = 7.9; *Figure 2B*). The funnelplot on the lower panel of *Figure 2A* shows the absence of selection bias, with all effect estimates falling within the 95% CI, and the risk of bias plot shows that the included trials were all high-quality (except for the CAPA trial that had one high risk of bias domain) for assessing all-cause mortality.

**Figure 3:**
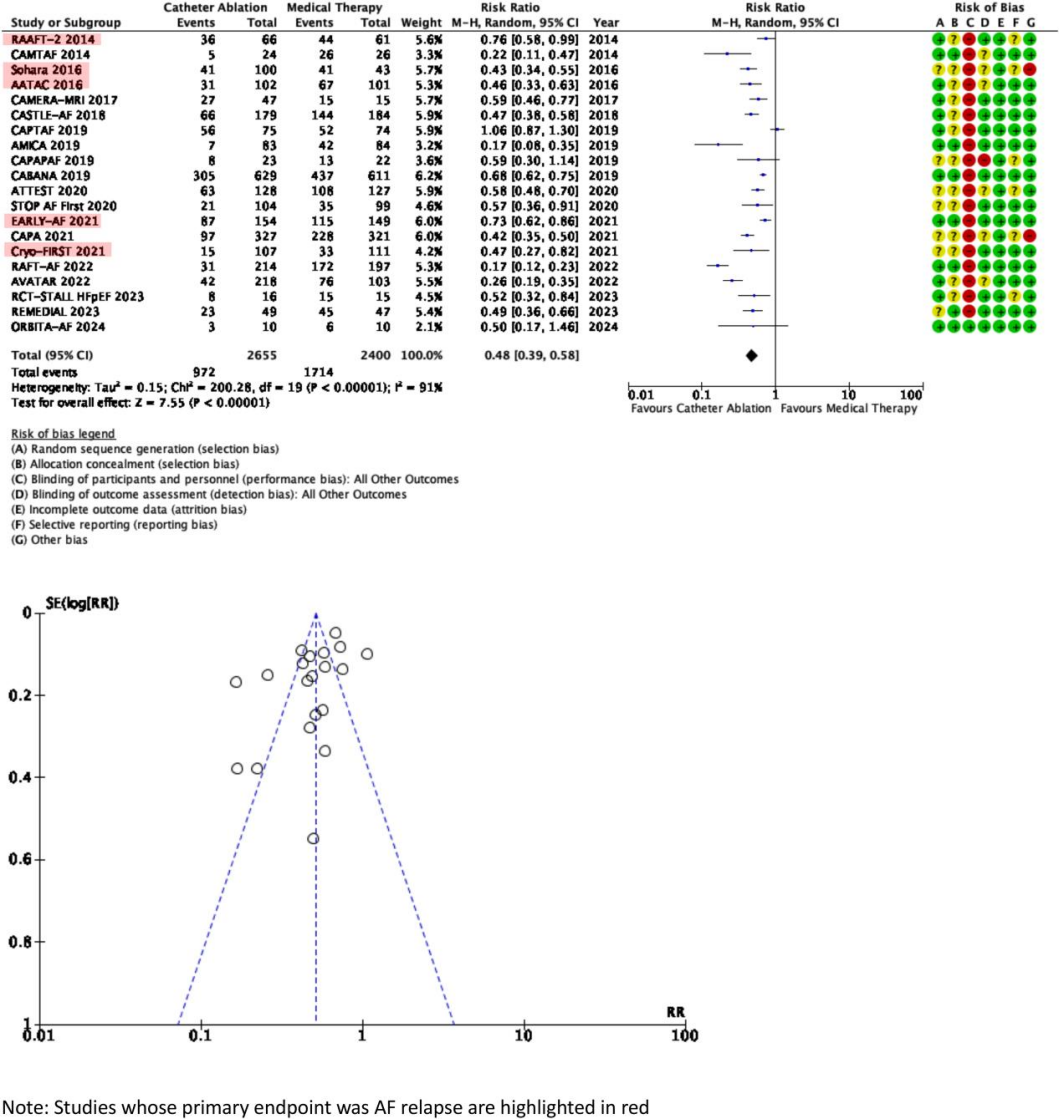
Analyses for atrial fibrillation relapse. Note: Studies whose primary endpoint was atrial fibrillation relapse are highlighted in red.

A pronounced reduction in AF relapse was also observed with catheter ablation (RR: 0.48, 95% CI 0.39–0.59, *P* < 0.00001, *I*^2^ = 91%, with 17 out of 20 trials showing a significant benefit; *Figure 3*), and funnelplots suggesting publication bias. A summary of the performed analyses is presented in *Table 2*.

**Table 2:** Summary of findings table

Outcome	Effect size 95% CI *P*	Studies Sample size	NNT to prevent one event	Heterogeneity Risk of bias assessment	Indirectness Imprecision Publication Bias	Interpretation Quality of evidence/GRADE
All-cause mortality	RR = 0.69 0.56–0.86 *P* = 0.0007	19 RCTs 6191 patients	44.7 patients 4.5% vs. 6.8%	Low heterogeneity (*I*^2^ = 2%) RoB—↓1 level (Selection)	No indirectness No imprecision No publication bias	Significant reduction in all-cause mortality Moderate quality 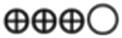
All-cause hospitalizations	RR = 0.57 0.39–0.85 *P* = 0.006	8 RCTs 1890 patients	7.9 patients 20.7% vs. 33.4%	High heterogeneity (*I*^2^ = 76%) RoB—↓1 level (Selection and performance)	No indirectness No imprecision NA (<10 RCT)	Significant reduction in all-cause hospitalizations Low quality 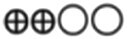
AF relapse	RR = 0.48 0.39–0.58 *P* < 0.00001	20 RCTs 5055 patients	2.9 patients 36.4% vs. 71.0%	High heterogeneity (*I*^2^ = 91%) RoB—↓1 level (Selection and performance)	No indirectness No imprecision Publication bias	Significant reduction in AF relapse Low quality 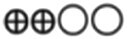
Cardiovascular mortality	RR = 0.55 0.34–0.87 *P* = 0.01	11 RCTs 4052 patients	50.0 patients 2.5% vs. 4.5%	Low heterogeneity (*I*^2^ = 33%) RoB—↓1 level (selection and performance)	No indirectness No imprecision No publication bias	Significant reduction in cardiovascular mortality Moderate quality 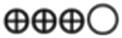
Cardiovascular hospitalizations	RR = 0.83 0.71–0.96 *P* = 0.01	7 RCTs 3589 patients	12.4 patients 35.6% vs. 43.7%	Low heterogeneity (*I*^2^ = 18%) RoB—↓1 level (Selection and performance)	No indirectness No imprecision NA (<10 RCT)	Significant reduction in cardiovascular hospitalizations Moderate quality 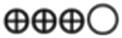
Heart failure hospitalizations	RR = 0.71 0.56–0.89 *P* = 0.004	5 RCTs 1638 patients	18.9 patients 13.4% vs. 18.7%	Low heterogeneity (*I*^2^ = 6%) RoB—↓1 level (selection and performance)	No indirectness No imprecision NA (<10 RCT)	Significant reduction in heart failure hospitalizations Moderate quality 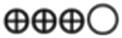
Change in AF burden	MD = 20.6 5.6–35.5 *P* = 0.007	3 RCTs 394 patients	—	High heterogeneity (*I*^2^ = 81%) RoB—↓1 level (selection)	No indirectness No imprecision NA (<10 RCT)	Significant reduction in AF burden Low quality 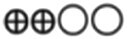
LVEF change	MD = 5.65 3.45–7.85 *P* < 0.00001	7 RCTs 1121 patients	—	High heterogeneity (*I*^2^ = 88%) RoB—↓1 level (selection and performance)	No indirectness No imprecision NA (<10 RCT)	Significant improvement in LVEF Low quality 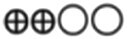
Quality of life MLHFQ △ over 12 months AFEQT △ over 12 months SF-36 at 12 months	MD = −7.96 −12.88 to −3.04 *P* = 0.002 MD = 6.98 4.80–9.17 *P* < 0.0001 MD = 3.54 1.23–5.84 *P* = 0.003	5 RCTs 653 patients 7 RCTs 3206 patients 4RCTs 2256 patients	—	Moderate heterogeneity (*I*^2^ = 68%) High heterogeneity (*I*^2^ = 96%) High heterogeneity (*I*^2^ = 67%) RoB—↓1 level (selection and performance)	No indirectness No imprecision NA (<10 RCT)	Significant improvement in quality of life Low quality 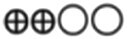

Compared to medical therapy, catheter ablation was also associated with a significant reduction cardiovascular mortality (RR: 0.55, 95% CI 0.34–0.87, *P* = 0.01; Supplementary material online, *Figure S1*), cardiovascular hospitalizations (RR: 0.83, 95% CI 0.71–0.96, *P* = 0.01; Supplementary material online, *Figure S2*), HF hospitalizations (RR: 0.71, 95% CI 0.56–0.89, *P* = 0.004, Supplementary material online, *Figure S3*), AF burden (MD: 20.6%, 95% CI 5.6–35.5%, *P* = 0.007; Supplementary material online, *Figure S4*), and led to an improvement in LVEF (MD: 5.7%, 95% CI 3.5–7.9%, *P* < 0.00001 Supplementary material online, *Figure S5*) and quality of life measures: MLHFQ (MD: −8.0, 95% CI −12.9% to −3.0, *P* = 0.002; Supplementary material online, *Figure S6*), AFEQT (MD: 7.0, 95% CI 4.8–9.2, *P* < 0.00001; Supplementary material online, *Figure S7*) and SF-36 (General Health—MD: 3.54, 95% CI 1.2–5.8; all other SF-36 parameters also in Supplementary material online, *Figure S8*).

All main analyses and plots in the manuscript are presented as random effects, as usually more conservative, and were also repeated using fixed effects models as highlighted in the methods section. This revealed no changes in the significance level or direction of effect, and the confidence intervals with the fixed effects model tended to be much narrower.

### Quality appraisal

A detailed description of the risk of bias assessment is provided in the Supplementary material online, *Table S6*. In sum, due to lack of personnel and patient blinding, all trials were considered high risk for that domain when objective outcomes were being considered (i.e. all outcomes except all-cause mortality, stroke, and AF burden). Besides this, most trials had good quality overall, with only four trials^26,31,37,38^ having high risk of bias for ≥2 domains (i.e. one further domain classified as high risk besides lack of personnel and patient blinding). One trial, ORBITA-AF,^35^ the first sham-controlled trial, was considered low risk for all domains.

GRADE assessments are presented in the summary of findings table (*Table 2*), alongside with the rationale for downgrading the quality of evidence. Quality was considered moderate for the outcomes: all-cause mortality, cardiovascular mortality, cardiovascular hospitalizations, and HF hospitalizations. All remaining assessments were considered low certainty.

### Sub-group and sensitivity analyses

All-cause mortality reduction was observed only for trials of patients with HF (see Supplementary material online, *Figure S9*), persistent AF, or a mix of paroxysmal and persistent AF patients. There was no mortality benefit from early ablation, as events (one in each arm) were observed for only one of the four trials.^25^

All-cause hospitalizations were reduced with catheter ablation among all assessed scenarios, with this effect being more pronounced in patients without HF, with paroxysmal AF, and also those receiving first-line ablation, with very low heterogeneity for all three scenarios (*I*^2^ = 0%).

Ablation reduced AF relapse by ≥50% when compared to medical therapy for most clinical scenarios: paroxysmal and persistent AF, presence/absence of HF, early ablation, or ablation after AAD failure. A more pronounced benefit was seen for persistent AF, HF, and failure of AAD (high heterogeneity for all three: *I*^2^ > 90%).

## Discussion

This systematic review provides clear up to date evidence on the benefit of catheter ablation vs. medical therapy for positively impacting hard endpoints and symptoms. All primary endpoints were significantly reduced by catheter ablation when compared to medical management: all-cause mortality, all-cause hospitalization, and AF relapse. A benefit vs. medical management was also demonstrated for all secondary endpoints: cardiovascular mortality, cardiovascular, and HF hospitalizations, AF burden (20.6% mean reduction), and LVEF recovery (5.7% mean improvement). Changes in quality of life met criteria for clinically meaningful changes with all utilized scales: MLHFQ (changes of ±5 points), AFEQT (changes of ±5 points), and SF-36 (a change of two points on the physical component summary and three points on the mental component summary).

The performed sensitivity analyses suggest that the mortality reduction benefit seems to be restricted to the HF population, and potentially more benefit may be expected in patients with persistent AF or more pronounced AF burden. Reduction in all-cause hospitalizations seems more pronounced for patients with paroxysmal AF, receiving first-line ablation, and with no history of HF. Finally, ablation is vastly more effective than medical therapy at reducing AF relapse across all assessed scenarios, including new-onset and drug-refractory AF, with a more pronounced benefit seen for persistent AF, and HF, settings where AAD are either less efficacious or may have detrimental side effects.

The observed benefit of catheter ablation on all-cause mortality requires some additional comment. CASTLE-AF, CASTLE-HTx, and AATAC all showed a significant reduction in mortality, and they all enrolled exclusively patients with HF with reduced LVEF, a population with known high annual mortality rate. These three studies only contributed with 35.6% (8.1% + 20.8% + 6.7%) of the weight for the final pooled analysis. CABANA and RAFT-AF contributed with 30.5% and 20.3%, respectively, and their effect estimates, 0.86 and 0.79, were situated on the side of benefit. RAFT-AF was a HF trial, and the mortality benefit in CABANA was driven by the HF population: in the ITT sub-analysis for HF patients in CABANA, a 43% relative reduction in all-cause mortality (HR 0.57, 95% CI 0.33–0.96) was observed with catheter ablation compared with drug therapy alone. These observations indicate that a mortality benefit with catheter ablation of AF may be expected in patients with HF, and our sub-analysis data does not confirm a mortality reduction benefit in the non-HF population (as the RR of 1.08 in *Table 3* seems to attest).

**Table 3:** Sub-analyses and sensitivity analyses

	*n* Trials, patients	All-cause mortality	All-cause hospital.	AF relapse
HF	8 2260	RR = 0.61 0.48–0.77 *P* < 0.0001 *I*^2^ = 0% NNT = 17.4	RR = 0.71 0.48–1.06 *P* = 0.10 *I*^2^ = 77%	RR = 0.38 0.25–0.58 *P* < 0.00001 *I*^2^ = 93% NNT = 2.3
No HF	14 4140	RR = 1.08 0.71–1.63 *P* = 0.72 *I*^2^ = 0%	RR = 0.38 0.25–0.59 *P* < 0.0001 *I*^2^ = 0% NNT = 8.7	RR = 0.56 0.45–0.68 *P* < 0.00001 *I*^2^ = 88% NNT = 3.3
Paroxysmal AF	7 1570	RR = 0.98 0.33–2.93 *P* = 0.97 *I*^2^ = 0%	RR = 0.38 0.25–0.60 *P* < 0.0001 *I*^2^ = 0% NNT = 8.6	RR = 0.52 0.39–0.69 *P* < 0.00001 *I*^2^ = 87% NNT = 3.3
Persistent AF	9 1787	RR = 0.72 0.51–1.02 *P* = 0.07 *I*^2^ = 0%	RR = 0.61 0.48–0.78 *P* < 0.0001 *I*^2^ = 0% NNT =7.7	RR = 0.33 0.22–0.50 *P* < 0.00001 *I*^2^ = 89% NNT = 2.0
Mixed parox/pers	6 3043	RR = 0.59 0.36–0.97 *P* = 0.04 *I*^2^ = 65% NNT = 32.6	RR = 0.96 0.83–1.12 *P* = 0.60 *I*^2^ = NA	RR = 0.62 0.47–0.83 *P* = 0.001 *I*^2^ = 89% NNT = 3.8
Early ablation	4 851	RR = 0.97 0.06–15.33 *P* = 0.98 *I*^2^ = NA	RR = 0.36 0.22–0.57 *P* < 0.0001 *I*^2^ = 0% NNT = 6.7	RR = 0.69 0.59–0.82 *P* < 0.00001 *I*^2^ = 20% NNT = 5.8
Ablation after AAD failure	18 5549	RR = 0.68 0.54–0.86 *P* = 0.002 *I*^2^ = 12% NNT = 38.2	RR = 0.72 0.50–1.04 *P* = 0.08 *I*^2^ = 69%	RR = 0.44 0.35–0.56 *P* < 0.00001 *I*^2^ = 92% NNT = 2.6
Higher quality studies (≤1 high risk of bias domain)	18 5360	RR = 0.68 0.54–0.86 *P* = 0.001 *I*^2^ = 10% NNT = 38.1	RR = 0.57 0.39–0.85 *P* = 0.006 *I*^2^ = 76% NNT = 7.9	RR = 0.48 0.39–0.60 *P* < 0.00001 *I*^2^ = 91% NNT = 3.0
Lower quality (≥2 high risk of bias domains)	4 1040	RR = 0.98 0.29–3.36 *P* = 0.98 *I*^2^ = NA	NA	RR = 0.43 0.37–0.49 *P* < 0.00001 *I*^2^ = 0% NNT = 2.5
Studies published <5 years ago	14 5448	RR = 0.79 0.62–1.00 *P* = 0.05 *I*^2^ = 0% NNT = 78.0	RR = 0.51 0.33–0.77 *P* = 0.002 *I*^2^ = 34% NNT = 9.6	RR = 0.45 0.34–0.61 *P* < 0.00001 *I*^2^ = 94% NNT = 2.9
Studies published ≥ 5 years ago	6 952	RR = 0.51 0.34–0.75 *P* = 0.0006 *I*^2^ = 0% NNT = 12.1	RR = 0.68 0.28–1.24 *P* = 0.21 *I*^2^ = 84%	RR = 0.50 0.40–0.62 *P* < 0.00001 *I*^2^ = 72% NNT = 2.6

The 2020 ESC guidelines propose AF ablation mainly as an option to deal with symptoms, with different classes of recommendation given for paroxysmal and persistent AF (IIa and IIb, respectively, prior to AAD, and Class I after failure of AAD). For patients with paroxysmal or persistent AF with HF and reduced LVEF, AF ablation is recommended as first option (Class I), or after failure of AAD (Class IIa), while a Class I recommendation is also present for HF with reduced LVEF population with the goal of improving LVEF, and Class IIa to improve survival and reduce HF hospitalizations.^7^

The 2023 ACC/AHA Guidelines provide a slightly different view, not using classification into paroxysmal or persistent AF to define recommendations, assigning Class I for ablation in cases of symptomatic drug-refractory AF, and new-onset AF in selected populations (young patients with symptomatic AF and few comorbidities).^8^ Furthermore, a Class I recommendation is present for early and aggressive rhythm control in cases of AF-induced cardiomyopathy with reduced LVEF, and for patients with AF and HF with reduced LVEF on guideline-directed medical therapy from whom ablation is expected to improve symptoms, quality of life, ventricular function, and cardiovascular outcomes.^8^

Currently, ongoing sham-controlled trials of AF ablation vs. medical therapy^35,48–50^ will ease concerns regarding lack of patient blinding in catheter ablation trials, and will provide further insight into the presence, if any, of a placebo effect for catheter ablation.

This systematic review presents up-to-date high-quality evidence to date to inform future guidelines on the impact of catheter ablation of AF for the reduction of AF relapse, AF burden, all-cause hospitalizations, and improvement in quality of life. Furthermore, for the HF population with reduced LVEF, we have shown recovery of LVEF and reduction in all-cause mortality, cardiovascular mortality, and HF hospitalizations.

## Conclusions

The findings of this systematic review of RCTs strongly suggest that the benefit of catheter ablation goes well beyond treating symptoms, as it also reduces hospitalizations and AF burden and improves quality of life. In addition, an impact on hard clinical outcomes, with an important mortality reduction and improvement in LVEF, was seen for patients with AF and HF. Physicians and patients should be aware of the favourable prognostic impact of catheter ablation when discussing treatment options.

## Supplementary Material

oeae058_Supplementary_Data

## Data Availability

No new data were generated or analysed in support of this research. All data were extracted from publications or provided by study authors and made available on the manuscript.

## Lead author biography

Rui Providencia is a Portuguese Consultant Cardiologist/Cardiac Electrophysiologist. After training in Portugal (Centro Hospitalar e Universitário de Coimbra and Hospital de Santa Cruz, Lisboa), he was an EP Fellow at Clinique Pasteur (Toulouse, France) and at The Heart Hospital and St. Bartholomew’s Hospital (London, UK). Currently, He is clinically active at St. Bartholomew’s Hospital and is a Professor of Cardiovascular Epidemiology and Evidence Synthesis at University College London. His main research interests are technology assessment, the epidemiology and heterogeneity of atrial fibrillation, and using genetics, omics, and electronic health records to identify novel drug targets and biomarkers.
