# Supplementary material for: Catheter ablation for atrial fibrillation and impact on clinical outcomes
Source: Eur Heart J Open. 2024 Jul 15;4(4):oeae058. doi: 10.1093/ehjopen/oeae058 (PMC11322836; doi:10.1093/ehjopen/oeae058)
Supplement: oeae058_Supplementary_Data [file oeae058_supplementary_data.pdf]

Supplementary Material

Supplementary Figure S1 – Analyses for Cardiovascular Mortality

Study or Subgroup	Catheter Ablation Events	Catheter Ablation Total	Medical Therapy Events	Medical Therapy Total	Weight	Risk Ratio M-H, Random, 95% CI	Year	A	B	C	D	E	F	G
CAMTAF 2014	0	25	1	24	2.1%	0.32 [0.01, 7.50]	2014	+	+	-	?	+	+	+
CASTLE-AF 2018	20	179	41	184	37.6%	0.50 [0.31, 0.82]	2018	+	?	-	+	+	+	+
AMICA 2019	3	98	6	100	10.0%	0.51 [0.13, 1.98]	2019	+	+	-	+	+	+	+
CABANA 2019	22	1108	23	1096	32.5%	0.95 [0.53, 1.69]	2019	+	+	-	+	+	+	+
STOP AF First 2020	0	104	0	99		Not estimable	2020	?	?	-	+	+	+	+
EARLY-AF 2021	0	154	0	149		Not estimable	2021	+	+	-	+	+	+	+
Cryo-FIRST 2021	0	107	0	111		Not estimable	2021	?	?	-	+	+	+	+
Ding 2022	0	102	0	102		Not estimable	2022	?	?	-	-	-	?	-
CASTLE-HTx 2023	5	97	19	97	17.8%	0.26 [0.10, 0.68]	2023	+	?	-	?	+	+	+
REMEDIAL 2023	0	49	0	47		Not estimable	2023	?	+	-	+	+	+	+
ORBITA-AF 2024	0	10	0	10		Not estimable	2024	+	+	+	+	+	+	+
Total (95% CI)		2033		2019	100.0%	0.55 [0.34, 0.87]								
Total events	50		90											

Heterogeneity: Tau² = 0.09; Chi² = 5.94, df = 4 (P = 0.20); I² = 33%

Test for overall effect: Z = 2.54 (P = 0.01)

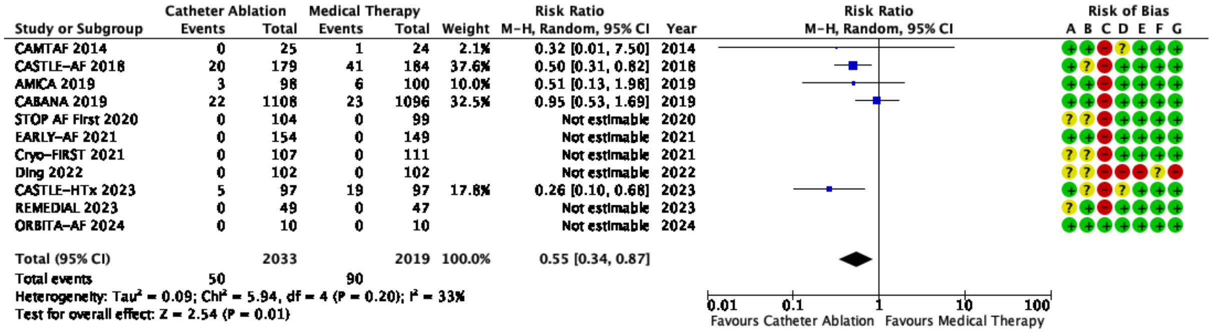

Risk of bias legend
(A) Random sequence generation (selection bias)
(B) Allocation concealment (selection bias)
(C) Blinding of participants and personnel (performance bias): All Other Outcomes
(D) Blinding of outcome assessment (detection bias): All Other Outcomes
(E) Incomplete outcome data (attrition bias)
(F) Selective reporting (reporting bias)
(G) Other bias

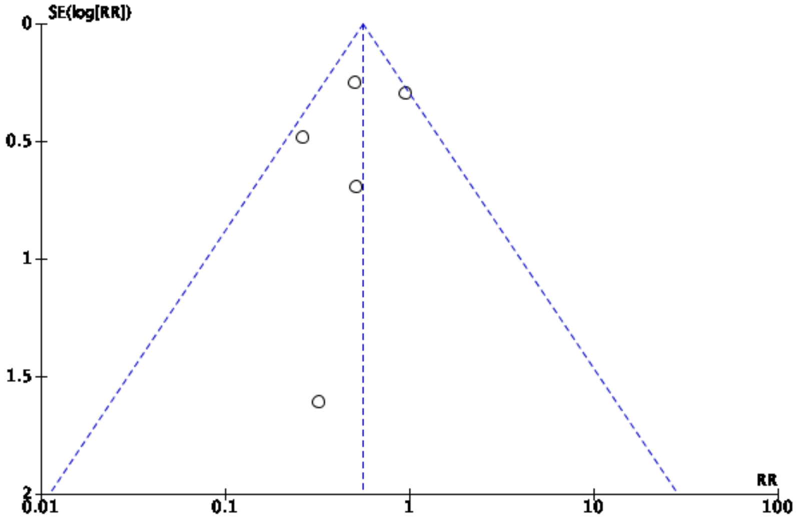

Supplementary Figure S2 – Analyses for CV Hospitalizations

	Catheter Ablation		Medical Therapy			Risk Ratio		Risk of Bias						
Study or Subgroup	Events	Total	Events	Total	Weight	M–H, Random, 95% CI	Year	A	B	C	D	E	F	G
CAMERA–MRI 2017	0	33	4	33	0.3%	0.11 [0.01, 1.98]	2017	+	?	−	+	+	+	+
CASTLE–AF 2018	64	179	89	184	24.6%	0.74 [0.58, 0.95]	2018	+	?	−	+	+	+	+
CABANA 2019	556	1108	605	1096	60.4%	0.91 [0.84, 0.98]	2019	+	+	−	+	+	+	+
RAFT–AF 2022	38	214	48	197	12.9%	0.73 [0.50, 1.06]	2022	+	+	−	+	+	+	+
AVATAR 2022	5	218	3	103	1.1%	0.79 [0.19, 3.23]	2022	+	?	−	?	+	+	+
Ding 2022	1	102	4	102	0.5%	0.25 [0.03, 2.20]	2022	?	?	−	−	−	?	−
ORBITA–AF 2024	0	10	1	10	0.2%	0.33 [0.02, 7.32]	2024	+	+	+	+	+	+	+
Total (95% CI)		1864		1725	100.0%	0.83 [0.71, 0.96]								
Total events	664		754											

Heterogeneity: Tau² = 0.01; Chi² = 7.36, df = 6 (P = 0.29); I² = 18%
Test for overall effect: Z = 2.48 (P = 0.01)

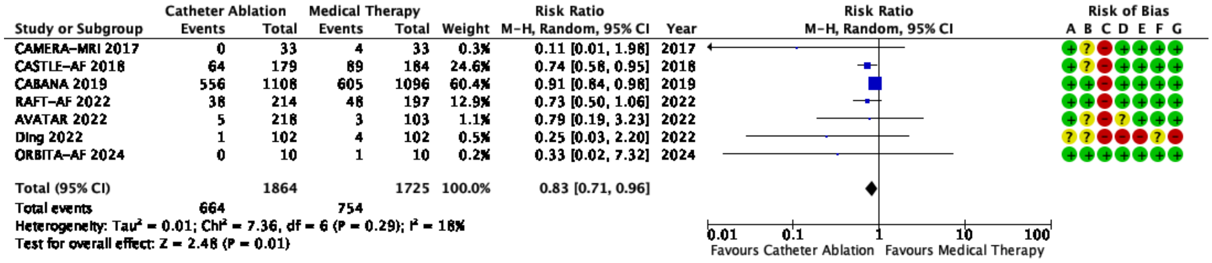

Risk of bias legend
(A) Random sequence generation (selection bias)
(B) Allocation concealment (selection bias)
(C) Blinding of participants and personnel (performance bias): All Other Outcomes
(D) Blinding of outcome assessment (detection bias): All Other Outcomes
(E) Incomplete outcome data (attrition bias)
(F) Selective reporting (reporting bias)
(G) Other bias

Supplementary Figure S3 – Analyses for Heart Failure hospitalizations

	Catheter Ablation		Medical Therapy			Risk Ratio		Risk of Bias						
Study or Subgroup	Events	Total	Events	Total	Weight	M–H, Random, 95% CI	Year	A	B	C	D	E	F	G
CAMERA–MRI 2017	0	33	2	33	0.6%	0.20 [0.01, 4.01]	2017	+	?	−	+	+	+	+
CASTLE–AF 2018	37	179	66	184	39.6%	0.58 [0.41, 0.81]	2018	+	?	−	+	+	+	+
CABANA 2019	34	378	37	400	25.4%	0.97 [0.62, 1.52]	2019	+	+	−	+	+	+	+
RAFT–AF 2022	38	214	48	197	33.8%	0.73 [0.50, 1.06]	2022	+	+	−	+	+	+	+
ORBITA–AF 2024	0	10	1	10	0.6%	0.33 [0.02, 7.32]	2024	+	+	+	+	+	+	+
Total (95% CI)		814		824	100.0%	0.71 [0.56, 0.89]								
Total events	109		154											

Heterogeneity: Tau² = 0.00; Chi² = 4.26, df = 4 (P = 0.37); I² = 6%
Test for overall effect: Z = 2.91 (P = 0.004)

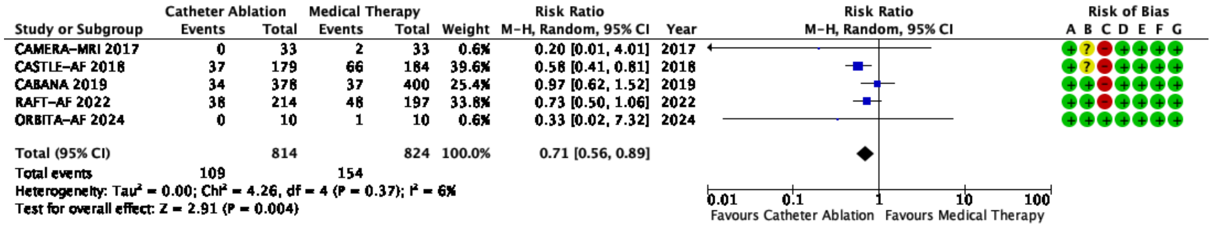

Risk of bias legend
(A) Random sequence generation (selection bias)
(B) Allocation concealment (selection bias)
(C) Blinding of participants and personnel (performance bias): All Other Outcomes
(D) Blinding of outcome assessment (detection bias): All Other Outcomes
(E) Incomplete outcome data (attrition bias)
(F) Selective reporting (reporting bias)
(G) Other bias

Supplementary Figure S4 – Analyses for AF Burden

Study or Subgroup	Catheter Ablation Mean	SD	Total	Medical Therapy Mean	SD	Total	Weight	Mean Difference IV, Random, 95% CI	Year
CASTLE–AF 2018	24	44	48	−13	45	36	25.1%	37.00 [17.74, 56.26]	2018
CAPTAF 2019	19.4	27.6	79	11.8	24.2	76	38.1%	7.60 [−0.56, 15.76]	2019
CASTLE–HTx 2023	31.4	33.3	89	8.6	26.3	66	36.8%	22.80 [13.41, 32.19]	2023
Total (95% CI)			216			178	100.0%	20.57 [5.61, 35.53]	

Heterogeneity: $Tau^2 = 135.39; Chi^2 = 10.64, df = 2 (P = 0.005); I^2 = 81\%$
Test for overall effect: $Z = 2.70 (P = 0.007)$

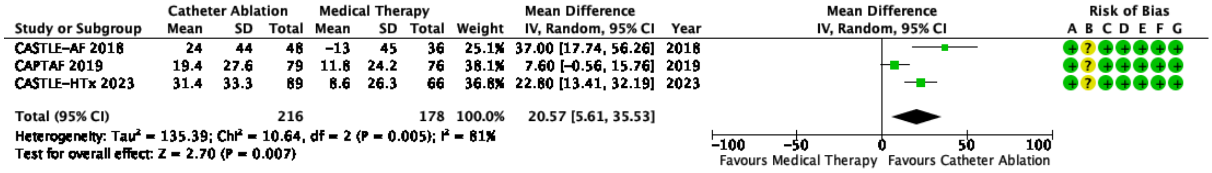

Risk of bias legend
(A) Random sequence generation (selection bias)
(B) Allocation concealment (selection bias)
(C) Blinding of participants and personnel (performance bias): All–Cause Mortality, Stroke & AF Burden
(D) Blinding of outcome assessment (detection bias): All–Cause Mortality, Stroke & AF Burden
(E) Incomplete outcome data (attrition bias)
(F) Selective reporting (reporting bias)
(G) Other bias

Supplementary Figure S5 – Analyses for LVEF change

Study or Subgroup	Catheter Ablation Mean	SD	Total	Medical Therapy Mean	SD	Total	Weight	Mean Difference IV, Random, 95% CI	Year
CAMTAF 2014	8.1	12.5	26	−3.6	9.7	24	7.9%	11.70 [5.52, 17.88]	2014
AATAC 2016	8.1	4	94	6.2	5	83	19.5%	1.90 [0.55, 3.25]	2016
CAMERA–MRI 2017	16.4	13.3	47	8.6	7.6	15	9.3%	7.80 [2.39, 13.21]	2017
CASTLE–AF 2018	8	8.5	50	0.2	9.6	37	12.7%	7.80 [3.91, 11.69]	2018
AMICA 2019	8.8	12.7	68	7.3	12.9	72	11.8%	1.50 [−2.74, 5.74]	2019
RAFT–AF 2022	10.1	1.2	214	3.8	1.2	197	21.0%	6.30 [6.07, 6.53]	2022
CASTLE–HTx 2023	7.8	7.6	97	1.4	7.2	97	17.7%	6.40 [4.32, 8.48]	2023
Total (95% CI)			596			525	100.0%	5.65 [3.45, 7.85]	

Heterogeneity: $Tau^2 = 5.96; Chi^2 = 48.68, df = 6 (P < 0.00001); I^2 = 88\%$
Test for overall effect: $Z = 5.04 (P < 0.00001)$

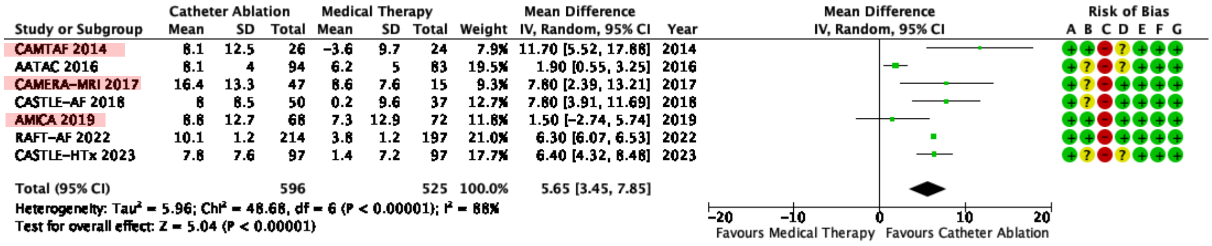

Risk of bias legend
(A) Random sequence generation (selection bias)
(B) Allocation concealment (selection bias)
(C) Blinding of participants and personnel (performance bias): All Other Outcomes
(D) Blinding of outcome assessment (detection bias): All Other Outcomes
(E) Incomplete outcome data (attrition bias)
(F) Selective reporting (reporting bias)
(G) Other bias

Note: Studies whose primary endpoint was AF relapse are highlighted in red

Supplementary Figure S6 – Analyses for Quality of Life - MLHFQ – mean change at 12 months or maximum available follow-up in the first year

Study or Subgroup	Catheter Ablation Mean	SD	Total	Medical Therapy Mean	SD	Total	Weight	Mean Difference IV, Random, 95% CI	Year
CAMTAF 2014	−18.3	25.6	25	0	26.6	23	8.5%	−18.30 [−33.09, −3.51]	2014
AATAC 2016	−11	19	94	−6	17	83	25.6%	−5.00 [−10.30, 0.30]	2016
AMICA 2019	−11.2	20	79	−8.9	24	78	21.2%	−2.30 [−9.22, 4.62]	2019
RAFT–AF 2022	−20.1	1.6	124	−13.9	1.7	116	36.4%	−6.20 [−6.62, −5.78]	2022
Chieng et al. 2023	−29.1	19.7	16	−0.4	22.7	15	8.3%	−28.70 [−43.70, −13.70]	2023
Total (95% CI)			338			315	100.0%	−7.96 [−12.88, −3.04]	

Heterogeneity: $Tau^2 = 17.28; Chi^2 = 12.63, df = 4 (P = 0.01); I^2 = 68\%$
Test for overall effect: $Z = 3.17 (P = 0.002)$

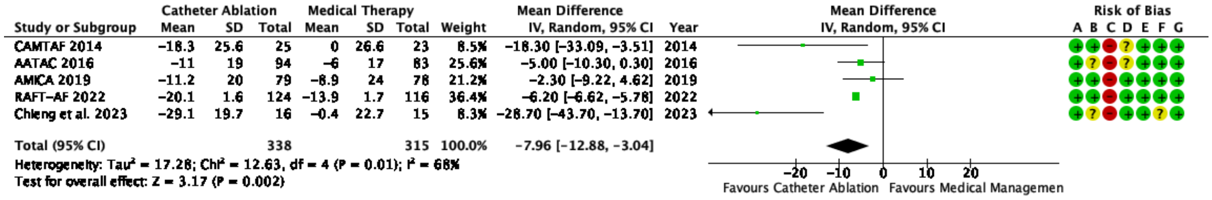

Risk of bias legend
(A) Random sequence generation (selection bias)
(B) Allocation concealment (selection bias)
(C) Blinding of participants and personnel (performance bias): All Other Outcomes
(D) Blinding of outcome assessment (detection bias): All Other Outcomes
(E) Incomplete outcome data (attrition bias)
(F) Selective reporting (reporting bias)
(G) Other bias

Note: Chieng et al. assessed QOL at 6 months

Supplementary Figure S7 – Analyses for Quality of Life - AFEQT – mean change at 12 months

Study or Subgroup	Catheter Ablation Mean	SD	Total	Medical Therapy Mean	SD	Total	Weight	Mean Difference IV, Random, 95% CI	Year	A	B	C	D	E	F	G
CABANA 2019	23.5	16.5	915	17.8	18.5	903	21.9%	5.70 [4.09, 7.31]	2019	+	+	−	+	+	+	+
STOP AF First 2020	33.3	20.3	90	21.5	24.2	90	7.7%	11.80 [5.27, 18.33]	2020	?	?	−	+	+	+	+
EARLY-AF 2021	26.9	1.9	154	22.9	2	149	24.6%	4.00 [3.56, 4.44]	2021	+	+	−	+	+	+	+
Cryo-FIRST 2021	26.9	15.2	89	18.2	20.2	92	10.3%	8.70 [3.50, 13.90]	2021	?	?	−	+	+	+	+
RAFT-AF 2022	23.4	1.5	214	16.1	1.6	197	24.7%	7.30 [7.00, 7.60]	2022	+	+	−	+	+	+	+
AVATAR 2022	30.1	25.6	194	21.8	22.8	88	8.7%	8.30 [2.33, 14.27]	2022	+	?	−	?	+	+	+
Chieng et al. 2023	29.9	20.4	16	9.2	21.9	15	2.0%	20.70 [5.78, 35.62]	2023	+	?	−	+	+	?	+
**Total (95% CI)**			1672			1534	100.0%	6.98 [4.80, 9.17]								

Heterogeneity: $Tau^2 = 5.00; Chi^2 = 155.70, df = 6 (P < 0.00001); I^2 = 96\%$
Test for overall effect: $Z = 6.26 (P < 0.00001)$

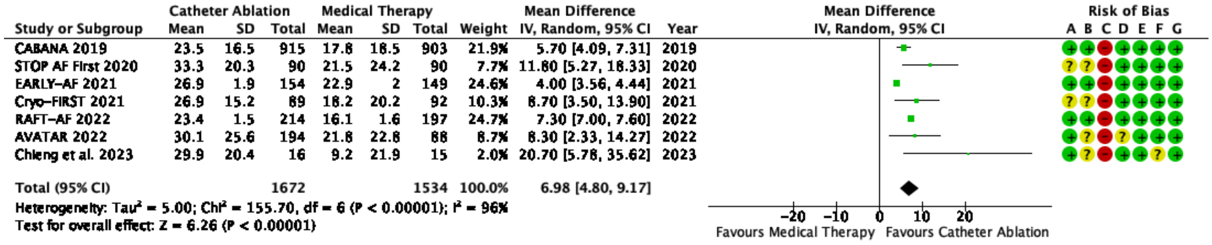

Risk of bias legend
(A) Random sequence generation (selection bias)
(B) Allocation concealment (selection bias)
(C) Blinding of participants and personnel (performance bias): All Other Outcomes
(D) Blinding of outcome assessment (detection bias): All Other Outcomes
(E) Incomplete outcome data (attrition bias)
(F) Selective reporting (reporting bias)
(G) Other bias

Supplementary Figure S8 – Analyses for Quality of Life – SF-36 - General Health 12 months

Study or Subgroup	Catheter Ablation Mean	SD	Total	Medical Therapy Mean	SD	Total	Weight	Mean Difference IV, Random, 95% CI	A	B	C	D	E	F	G
CABANA 2019	6.5	18	909	3	17.8	869	32.3%	3.50 [1.85, 5.15]	+	+	−	+	+	+	+
CAPTAF 2019	11.9	13.4	74	3.1	12.5	72	17.0%	8.80 [4.60, 13.00]	+	?	−	+	+	+	+
Sohara 2016	2.4	7	100	0.3	6.79	32	25.2%	2.10 [−0.62, 4.82]	?	?	−	?	+	?	−
Cryo-FIRST 2021	52.8	9.1	90	51.3	9.2	90	25.5%	1.50 [−1.17, 4.17]	?	?	−	+	+	+	+
**Total (95% CI)**			1173			1083	100.0%	3.54 [1.23, 5.84]							

Heterogeneity: $Tau^2 = 3.57; Chi^2 = 9.12, df = 3 (P = 0.03); I^2 = 67\%$
Test for overall effect: $Z = 3.00 (P = 0.003)$

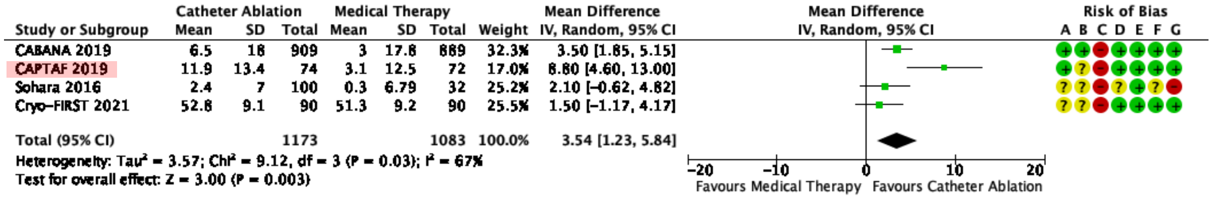

Risk of bias legend
(A) Random sequence generation (selection bias)
(B) Allocation concealment (selection bias)
(C) Blinding of participants and personnel (performance bias): All Other Outcomes
(D) Blinding of outcome assessment (detection bias): All Other Outcomes
(E) Incomplete outcome data (attrition bias)
(F) Selective reporting (reporting bias)
(G) Other bias

Note: Studies whose primary endpoint was AF relapse are highlighted in red

Physical Functioning 12 months

Study or Subgroup	Catheter Ablation Mean	SD	Total	Medical Therapy Mean	SD	Total	Weight	Mean Difference IV, Random, 95% CI	A	B	C	D	E	F	G
CABANA 2019	9.4	18.5	917	5.4	18.3	895	49.6%	4.00 [2.31, 5.69]	+	+	−	+	+	+	+
CAPTAF 2019	10.2	12.1	74	4.1	10.6	71	10.4%	6.10 [2.40, 9.80]	+	?	−	+	+	+	+
Cryo-FIRST 2021	54.1	6.4	90	51	8.4	90	29.9%	3.10 [0.92, 5.28]	?	?	−	+	+	+	+
Sohara 2016	1.9	9	100	−1.3	9.62	32	10.0%	3.20 [−0.57, 6.97]	?	?	−	?	+	?	−
**Total (95% CI)**			1181			1088	100.0%	3.87 [2.68, 5.06]							

Heterogeneity: $Tau^2 = 0.00; Chi^2 = 2.02, df = 3 (P = 0.57); I^2 = 0\%$
Test for overall effect: $Z = 6.35 (P < 0.00001)$

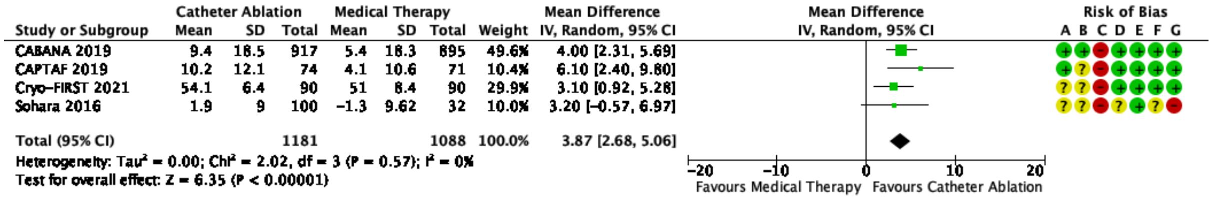

Risk of bias legend
(A) Random sequence generation (selection bias)
(B) Allocation concealment (selection bias)
(C) Blinding of participants and personnel (performance bias): All Other Outcomes
(D) Blinding of outcome assessment (detection bias): All Other Outcomes
(E) Incomplete outcome data (attrition bias)
(F) Selective reporting (reporting bias)
(G) Other bias

Role Physical 12 months

Study or Subgroup	Catheter Ablation Mean	SD	Total	Medical Therapy Mean	SD	Total	Weight	Mean Difference IV, Random, 95% CI	A	B	C	D	E	F	G
CABANA 2019	17.9	26.2	901	12.8	27.3	880	36.1%	5.10 [2.61, 7.59]	+	+	−	+	+	+	+
CAPTAF 2019	17.9	30.6	74	4.4	31.9	72	5.2%	13.50 [3.36, 23.64]	+	?	−	+	+	+	+
Sohara 2016	3	10	100	−1.5	10.2	32	22.3%	4.50 [0.46, 8.54]	?	?	−	?	+	?	−
Cryo-FIRST 2021	50.7	7.5	90	48.2	9.2	90	36.4%	2.50 [0.05, 4.95]	?	?	−	+	+	+	+
**Total (95% CI)**			1165			1074	100.0%	4.46 [2.03, 6.89]							

Heterogeneity: $Tau^2 = 2.65; Chi^2 = 5.59, df = 3 (P = 0.13); I^2 = 46\%$
Test for overall effect: $Z = 3.60 (P = 0.0003)$

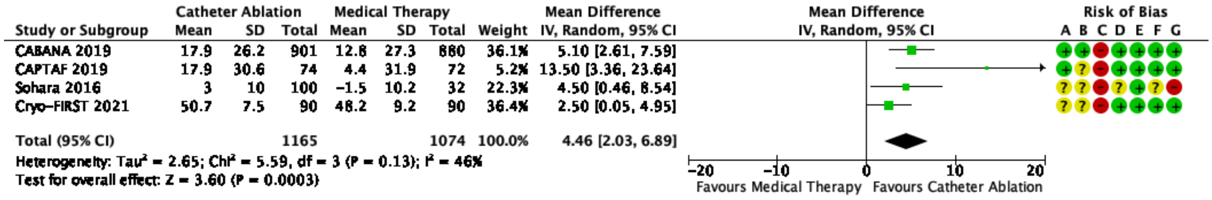

Risk of bias legend
(A) Random sequence generation (selection bias)
(B) Allocation concealment (selection bias)
(C) Blinding of participants and personnel (performance bias): All Other Outcomes
(D) Blinding of outcome assessment (detection bias): All Other Outcomes
(E) Incomplete outcome data (attrition bias)
(F) Selective reporting (reporting bias)
(G) Other bias

Bodily Pain 12 months

Study or Subgroup	Catheter Ablation Mean	SD	Total	Medical Therapy Mean	SD	Total	Weight	Mean Difference IV, Random, 95% CI	Risk of Bias A	B	C	D	E	F	G
CABANA 2019	5.7	19.2	912	2.8	19.3	891	59.2%	2.90 [1.12, 4.68]	+	+	-	+	+	+	+
CAPTAF 2019	5.7	21	74	3.8	17.5	72	4.8%	1.90 [-4.36, 8.16]	+	?	-	+	+	+	+
Sohara 2016	1.1	9	100	-0.4	9.62	32	13.2%	1.50 [-2.27, 5.27]	?	?	-	?	+	?	-
Cryo-FIRST 2021	54	8.8	90	52.2	10.7	90	22.8%	1.80 [-1.06, 4.66]	?	?	-	+	+	+	+
**Total (95% CI)**			1176			1085	100.0%	2.42 [1.05, 3.78]							

Heterogeneity: Tau² = 0.00; Chi² = 0.72, df = 3 (P = 0.87); I² = 0%
Test for overall effect: Z = 3.46 (P = 0.0005)

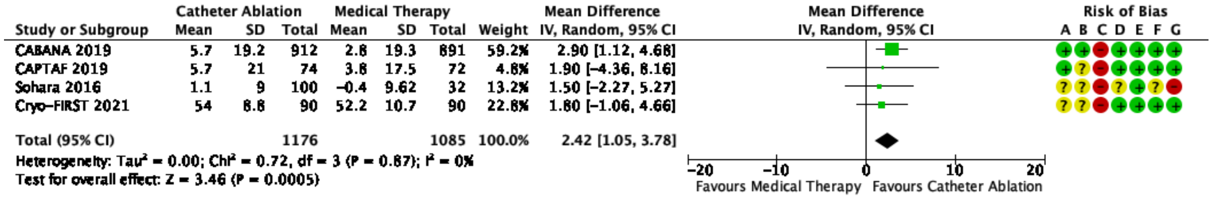

Risk of bias legend
(A) Random sequence generation (selection bias)
(B) Allocation concealment (selection bias)
(C) Blinding of participants and personnel (performance bias): All Other Outcomes
(D) Blinding of outcome assessment (detection bias): All Other Outcomes
(E) Incomplete outcome data (attrition bias)
(F) Selective reporting (reporting bias)
(G) Other bias

Mental Health 12 months

Study or Subgroup	Catheter Ablation Mean	SD	Total	Medical Therapy Mean	SD	Total	Weight	Mean Difference IV, Random, 95% CI	Risk of Bias A	B	C	D	E	F	G
CABANA 2019	9.9	13.8	906	6.4	12.8	888	69.3%	3.50 [2.27, 4.73]	+	+	-	+	+	+	+
CAPTAF 2019	6.1	10.4	74	0.8	9.8	72	9.8%	5.30 [2.02, 8.58]	+	?	-	+	+	+	+
Cryo-FIRST 2021	50.3	9.4	90	48.6	10.2	90	12.8%	1.70 [-1.17, 4.57]	?	?	-	+	+	+	+
Sohara 2016	2.7	9	100	-1	9.05	32	8.1%	3.70 [0.10, 7.30]	?	?	-	?	+	?	-
**Total (95% CI)**			1170			1082	100.0%	3.46 [2.44, 4.49]							

Heterogeneity: Tau² = 0.00; Chi² = 2.68, df = 3 (P = 0.44); I² = 0%
Test for overall effect: Z = 6.62 (P < 0.00001)

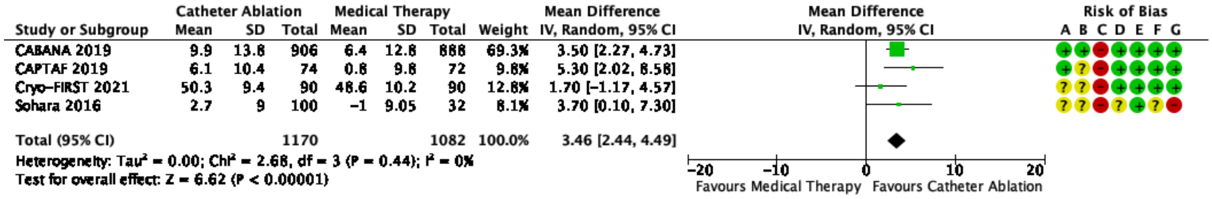

Risk of bias legend
(A) Random sequence generation (selection bias)
(B) Allocation concealment (selection bias)
(C) Blinding of participants and personnel (performance bias): All Other Outcomes
(D) Blinding of outcome assessment (detection bias): All Other Outcomes
(E) Incomplete outcome data (attrition bias)
(F) Selective reporting (reporting bias)
(G) Other bias

Social Functioning 12 months

Study or Subgroup	Catheter Ablation Mean	SD	Total	Medical Therapy Mean	SD	Total	Weight	Mean Difference IV, Random, 95% CI	Risk of Bias A	B	C	D	E	F	G
CABANA 2019	15.9	16.7	916	11.3	17.2	896	61.0%	4.60 [3.04, 6.16]	+	+	-	+	+	+	+
CAPTAF 2019	13.5	18.9	74	6.3	17	72	4.4%	7.20 [1.37, 13.03]	+	?	-	+	+	+	+
Sohara 2016	2.5	10	100	-0.1	10.2	32	9.1%	2.60 [-1.44, 6.64]	?	?	-	?	+	?	-
Cryo-FIRST 2021	51.3	7.1	90	47.7	9.3	90	25.5%	3.60 [1.18, 6.02]	?	?	-	+	+	+	+
**Total (95% CI)**			1180			1090	100.0%	4.28 [3.06, 5.50]							

Heterogeneity: Tau² = 0.00; Chi² = 2.09, df = 3 (P = 0.55); I² = 0%
Test for overall effect: Z = 6.87 (P < 0.00001)

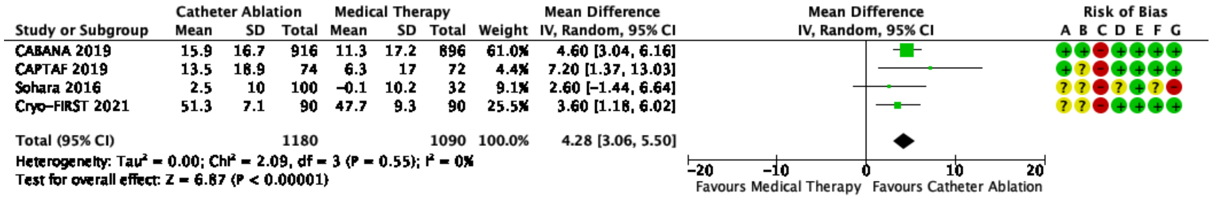

Risk of bias legend
(A) Random sequence generation (selection bias)
(B) Allocation concealment (selection bias)
(C) Blinding of participants and personnel (performance bias): All Other Outcomes
(D) Blinding of outcome assessment (detection bias): All Other Outcomes
(E) Incomplete outcome data (attrition bias)
(F) Selective reporting (reporting bias)
(G) Other bias

Role Emotional 12 months

Study or Subgroup	Catheter Ablation Mean	SD	Total	Medical Therapy Mean	SD	Total	Weight	Mean Difference IV, Random, 95% CI	Risk of Bias A	B	C	D	E	F	G
CABANA 2019	13.3	21.6	897	10	22.6	883	37.9%	3.30 [1.25, 5.35]	+	+	-	+	+	+	+
CAPTAF 2019	17.9	26.3	74	4.4	24.8	72	7.0%	13.50 [5.21, 21.79]	+	?	-	+	+	+	+
Sohara 2016	3.4	9	100	-0.9	8.5	32	25.0%	4.30 [0.87, 7.73]	?	?	-	?	+	?	-
Cryo-FIRST 2021	49.4	8.7	90	46.6	10.7	90	30.0%	2.80 [-0.05, 5.65]	?	?	-	+	+	+	+
**Total (95% CI)**			1161			1077	100.0%	4.12 [1.76, 6.48]							

Heterogeneity: Tau² = 2.72; Chi² = 6.01, df = 3 (P = 0.11); I² = 50%
Test for overall effect: Z = 3.42 (P = 0.0006)

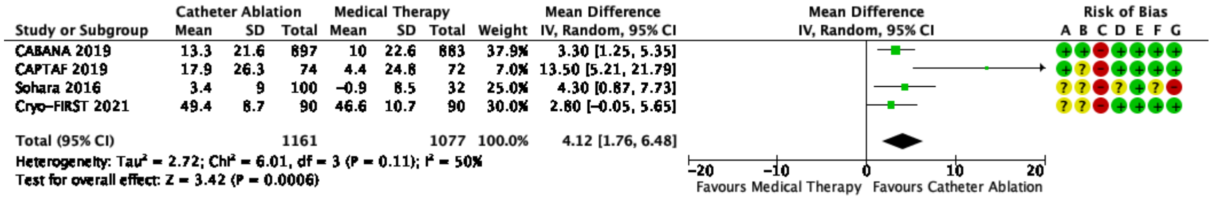

Risk of bias legend
(A) Random sequence generation (selection bias)
(B) Allocation concealment (selection bias)
(C) Blinding of participants and personnel (performance bias): All Other Outcomes
(D) Blinding of outcome assessment (detection bias): All Other Outcomes
(E) Incomplete outcome data (attrition bias)
(F) Selective reporting (reporting bias)
(G) Other bias

Vitality 12 months

Study or Subgroup	Catheter Ablation Mean	Catheter Ablation SD	Catheter Ablation Total	Medical Therapy Mean	Medical Therapy SD	Medical Therapy Total	Weight	Mean Difference IV, Random, 95% CI
CABANA 2019	13	16.8	906	9.6	16.4	888	36.1%	3.40 [1.86, 4.94]
CAPTAF 2019	14	14.8	74	4.6	12.8	72	16.7%	9.40 [4.92, 13.88]
Sohara 2016	2.1	10	100	−0.1	9.62	32	19.8%	2.20 [−1.67, 6.07]
Cryo−FIRST 2021	54.5	8.7	90	52.2	9.5	90	27.5%	2.30 [−0.36, 4.96]
Total (95% CI)			1170			1082	100.0%	3.86 [1.51, 6.20]

Heterogeneity: Tau² = 3.36; Chi² = 7.88, df = 3 (P = 0.05); I² = 62%
Test for overall effect: Z = 3.23 (P = 0.001)

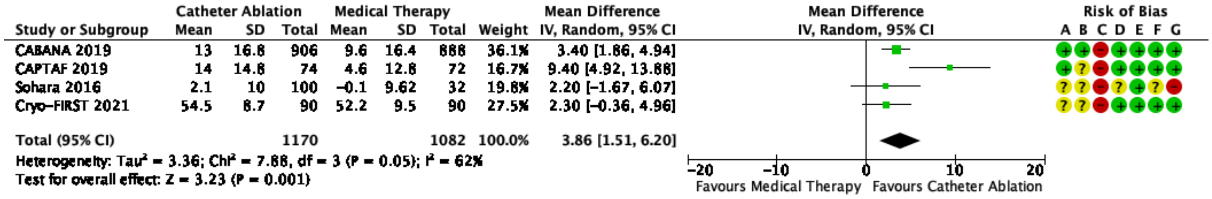

Risk of bias legend
(A) Random sequence generation (selection bias)
(B) Allocation concealment (selection bias)
(C) Blinding of participants and personnel (performance bias): All Other Outcomes
(D) Blinding of outcome assessment (detection bias): All Other Outcomes
(E) Incomplete outcome data (attrition bias)
(F) Selective reporting (reporting bias)
(G) Other bias

Supplementary Figure S9 – Sub-analysis for all-cause mortality in heart failure trials

Study or Subgroup	Catheter Ablation Events	Catheter Ablation Total	Medical Therapy Events	Medical Therapy Total	Weight	Risk Ratio M−H, Random, 95% CI	Year
CAMTAF 2014	0	26	1	24	0.6%	0.31 [0.01, 7.23]	2014
AATAC 2016	8	102	18	101	9.0%	0.44 [0.20, 0.97]	2016
CAMERA−MRI 2017	0	33	0	33		Not estimable	2017
CASTLE−AF 2018	24	179	46	184	27.7%	0.54 [0.34, 0.84]	2018
AMICA 2019	8	100	8	95	6.3%	0.95 [0.37, 2.43]	2019
CABANA−HF−Cohort	23	378	37	400	22.2%	0.66 [0.40, 1.09]	2021
RAFT−AF 2022	29	214	34	197	26.8%	0.79 [0.50, 1.24]	2022
CASTLE−HTx 2023	6	97	19	97	7.3%	0.32 [0.13, 0.76]	2023
Total (95% CI)		1129		1131	100.0%	0.61 [0.48, 0.77]	
Total events	98		163				

Heterogeneity: Tau² = 0.00; Chi² = 5.47, df = 6 (P = 0.49); I² = 0%
Test for overall effect: Z = 4.14 (P < 0.0001)

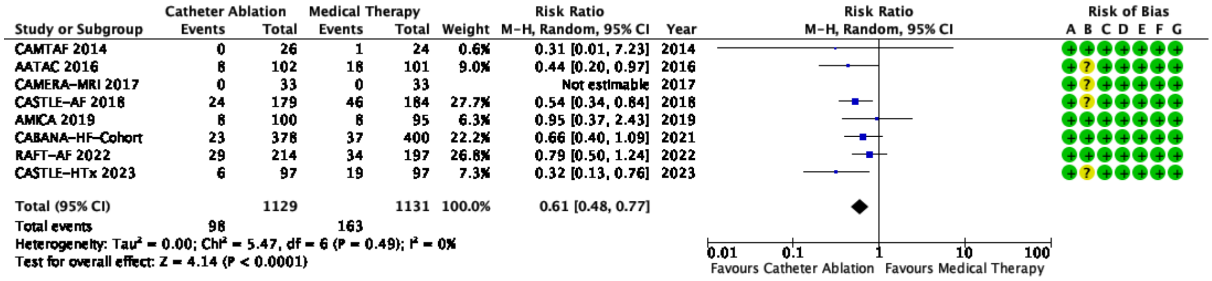

Risk of bias legend
(A) Random sequence generation (selection bias)
(B) Allocation concealment (selection bias)
(C) Blinding of participants and personnel (performance bias): All−Cause Mortality, Stroke & AF Burden
(D) Blinding of outcome assessment (detection bias): All−Cause Mortality, Stroke & AF Burden
(E) Incomplete outcome data (attrition bias)
(F) Selective reporting (reporting bias)
(G) Other bias

Supplementary Table 1. Excluded Studies

Trial Registration or PMID	Year	Title	Reason	Source
NCT02341105	2015	AMIOdarone vs. Catheter Ablation for Prevention of Recurrent Symptomatic Atrial Fibrillation	Protocol of study that was terminated due to low recruitment rate	https://clinicaltrials.gov/show/NCT02341105
ISRCTN46898887	2015	Catheter ablation for the cure of atrial fibrillation study	Wrong intervention - catheter ablation + AADs vs AADs, and study > 10 years - ablated between 2002 and 2003.	https://trialsearch.who.int/Trial2.aspx?TrialID=ISRCTN46898887
NCT05559073	2022	Outcomes of Early Referral to Radiofrequency Ablation in Symptomatic Atrial Fibrillation Patients	wrong comparator - deferred ablation	https://clinicaltrials.gov/show/NCT05559073
NCT03410966	2017	Atrial Fibrillation Ablation	Protocol of study marked as "complete" but results not published	https://clinicaltrials.gov/show/NCT03410966
NCT02285387	2014	Rhythm Control of AF in Patients With Acute Stroke	wrong intervention - rhythm control with drugs - RAFAS study	https://clinicaltrials.gov/show/NCT02285387
NCT04664686	2020	Rate or Rhythm Control in CRT: the RHYTHMIC Study	wrong comparator - AV node ablation	https://clinicaltrials.gov/show/NCT04664686
NCT04906668	2021	ABLATE Versus PACE: PVI or AV Node Ablation and PM Implantation for Elderly Patients With Persistent AF	Wrong comparator - AV node ablation	https://clinicaltrials.gov/show/NCT04906668
NCT02686749	2016	Catheter Ablation vs. Medical Therapy in Congested Hearts With AF	protocol of study marked as "complete" but results not published	https://clinicaltrials.gov/show/NCT02686749
NCT02509754	2015	Atrial Fibrillation Ablation Compared to Rate Control Strategy in Patients With Impaired Left Ventricular Function	protocol - terminated trial? no updates since 2015. Estimated completion date in 2018	https://clinicaltrials.gov/show/NCT02509754
35277805	2022	Catheter Ablation vs. Antiarrhythmic Drugs as Therapy for Paroxysmal Atrial Fibrillation.	Wrong design - review paper	https://dx.doi.org/10.1007/s11606-021-07211-8
	2022	STROKE RISK IN PATIENTS WITH PAROXYSMAL AND PERSISTENT ATRIAL FIBRILLATION TREATED WITH ANTI-ARRHYTHMIC DRUGS VS. CATHETER ABLATION.	Wrong design: not a RCT	https://dx.doi.org/10.1016/S0735-1097%2820%2930962-1
31082538	2019	Five-year outcomes in cardiac surgery patients with atrial fibrillation undergoing concomitant surgical ablation versus no ablation. The long-term follow-up of the PRAGUE-12 Study.	wrong intervention – concomitant surgical ablation	https://dx.doi.org/10.1016/j.hrthm.2019.05.001
	2019	Stroke risk in paroxysmal and persistent atrial fibrillation treated with anti-arrhythmic drugs vs catheter ablation.	Wrong study design: not a RCT	https://dx.doi.org/10.1002/joa3.12267
	2019	Stroke risk in paroxysmal and persistent atrial fibrillation treated with anti-arrhythmic drugs vs catheter ablation.	Wrong study design: not a RCT	https://dx.doi.org/10.1161/circ.140.suppl_1.10717
27566295	2016	Long-term efficacy of catheter ablation as first-line therapy for paroxysmal atrial fibrillation: 5-year outcome in a randomised clinical trial.	study published > 10 years ago - MANTRA-PAF	https://dx.doi.org/10.1136/heartjnl-2016-309781

24135832	2013	Catheter ablation vs. antiarrhythmic drug treatment of persistent atrial fibrillation: A multicentre, randomized, controlled trial (SARA study).	published > 10 years ago	https://dx.doi.org/10.1093/eurheartj/eht457
35348642	2022	Five-year results of Amaze: a randomized controlled trial of adjunct surgery for atrial fibrillation.	wrong intervention - surgical ablation	https://dx.doi.org/10.1093/ejcts/ezac181
	2020	Early Rhythm-Control Therapy in Patients with Atrial Fibrillation.	wrong intervention - EAST AFNET-4 early rhythm control including drugs and minority of ablation patients	https://dx.doi.org/10.1056/NEJMoa2019422

Supplementary Table 2. Additional Reports from Included Trials

PMID	Title	Comment	Source
36891899	Economic and Health Value of Delaying Atrial Fibrillation Progression Using Radiofrequency Catheter Ablation.	ATTEST substudy	https://dx.doi.org/10.1161/CIRCEP.122.011237
32018203	Cryoballoon catheter ablation versus antiarrhythmic drugs as a first-line therapy for patients with paroxysmal atrial fibrillation: Rationale and design of the international Cryo-FIRST study.	CRYO-First Protocol	https://dx.doi.org/10.1016/j.ahj.2019.12.006
36342178	Progression of Atrial Fibrillation after Cryoablation or Drug Therapy.	EARLY-AF - longterm follow-up	https://dx.doi.org/10.1056/NEJMoa2212540
36688367	Effects of Ablation Versus Drug Therapy on Quality of Life by Sex in Atrial Fibrillation: Results From the CABANA Trial.	CABANA substudy	https://dx.doi.org/10.1161/JAHA.122.027871
35726631	Cost-Effectiveness of Catheter Ablation Versus Antiarrhythmic Drug Therapy in Atrial Fibrillation: The CABANA Randomized Clinical Trial.	CABANA substudy	https://dx.doi.org/10.1161/CIRCULATIONAHA.122.058575
34933570	Association between Age and Outcomes of Catheter Ablation Versus Medical Therapy for Atrial Fibrillation: Results from the CABANA Trial.	CABANA substudy	https://dx.doi.org/10.1161/CIRCULATIONAHA.121.055297
34666139	Quality of life after the initial treatment of atrial fibrillation with cryoablation versus drug therapy.	STOP-AF First substudy	https://dx.doi.org/10.1016/j.hrthm.2021.10.009
34508694	Initial rhythm control with cryoballoon ablation vs drug therapy: Impact on quality of life and symptoms.	CRYO-FIRST substudy	https://dx.doi.org/10.1016/j.ahj.2021.08.007
33848199	Treatment-Related Changes in Left Atrial Structure in Atrial Fibrillation: Findings From the CABANA Imaging Substudy.	CABANA substudy	https://dx.doi.org/10.1161/CIRCEP.120.008540
33554614	Ablation Versus Drug Therapy for Atrial Fibrillation in Heart Failure: Results From the CABANA Trial.	CABANA substudy	https://dx.doi.org/10.1161/CIRCULATIONAHA.120.050991
34238436	Ablation Versus Drug Therapy for Atrial Fibrillation in Racial and Ethnic Minorities.	CABANA substudy	https://dx.doi.org/10.1016/j.jacc.2021.04.092
34223671	The results of health-related quality of life assessment depend on the prevailing rhythm at the assessment: Experience from the CAPTAF trial (Catheter Ablation Compared with Pharmacological Therapy for Atrial Fibrillation).	CAPTAF substudy	https://dx.doi.org/10.1111/jce.15147
33472052	A randomized ablation-based atrial fibrillation rhythm control versus rate control trial in patients with heart failure and high burden atrial fibrillation: The RAFT-AF trial rationale and design.	RAFT-AF Protocol	https://dx.doi.org/10.1016/j.ahj.2021.01.012
	B-PO02-086 METHODS AND OUTCOMES FOR ABLATION IN THE CABANA TRIAL.	CABANA substudy	https://dx.doi.org/10.1016/j.hrthm.2021.06.340
32903044	Impact of Left Ventricular Function and Heart Failure Symptoms on Outcomes Post Ablation of Atrial Fibrillation in Heart Failure: CASTLE-AF Trial.	CASTLE-AF substudy	https://dx.doi.org/10.1161/CIRCEP.120.008461
33334453	Catheter Ablation Versus Medication in Atrial Fibrillation and Systolic Dysfunction: Late Outcomes of CAMERA-MRI Study.	CAMERA-MRI - longterm outcomes	https://dx.doi.org/10.1016/j.jacep.2020.08.019
32586583	Recurrence of Atrial Fibrillation After Catheter Ablation or Antiarrhythmic Drug Therapy in the CABANA Trial.	CABANA substudy	https://dx.doi.org/10.1016/j.jacc.2020.04.065
	Cryoballoon Catheter Ablation versus Antiarrhythmic Drug as a First-Line Therapy for Patients with Paroxysmal Atrial Fibrillation: Results of the Cryo-FIRST Study.	CRYO-FIRST presentation	https://dx.doi.org/10.1161/circ.142.suppl_3.13915
	Impact of treatment strategies for AF on the progression and regression of AF type in the CABANA trial.	CABANA sub-study	https://dx.doi.org/10.1093/ehjci/ehaa946.0680
	CATHETER ABLATION OF ATRIAL FIBRILLATION ON HEART FAILURE SYMPTOMS AND QUALITY OF LIFE.	CASTLE-AF substudy	https://dx.doi.org/10.1016/j.hrthm.2019.04.018

	ASSOCIATION BETWEEN IMPROVEMENT IN LEFT VENTRICULAR FUNCTION, MORTALITY AND HOSPITALIZATION POST ABLATION OF ATRIAL FIBRILLATION IN PATIENTS WITH HEART FAILURE -THE CASTLE-AF TRIAL.	CASTLE-AF substudy	https://dx.doi.org/10.1016/j.hrthm.2019.04.017
30139501	Regression of Diffuse Ventricular Fibrosis Following Restoration of Sinus Rhythm With Catheter Ablation in Patients With Atrial Fibrillation and Systolic Dysfunction: A Substudy of the CAMERA MRI Trial.	CAMERA-MRI substudy	https://dx.doi.org/10.1016/j.jacep.2018.04.013
29754661	Catheter Ablation versus Antiarrhythmic Drug Therapy for Atrial Fibrillation (CABANA) Trial: Study Rationale and Design.	CABANA protocol	https://dx.doi.org/10.1016/j.ahj.2018.02.015
30342299	A randomized clinical trial of early invasive intervention for atrial fibrillation (EARLY-AF) - methods and rationale.	EARLY-AF protocol	https://dx.doi.org/10.1016/j.ahj.2018.05.020
	Catheter ablation vs. antiarrhythmic drug therapy for atrial fibrillation: The results of the cabana multicenter international randomized clinical trial.	CABANA presentation at HRS	
	Pulmonary vein isolation alone is not superior to amiodarone for the treatment of persistent atrial fibrillation in patients with congestive heart failure and an implanted device: Results from the AATAC randomized trial.	AATAC trial presentation	
35640922	Influence of atrial fibrillation type on outcomes of ablation vs. drug therapy: results from CABANA.	CABANA substudy	https://dx.doi.org/10.1093/europace/euac055
31221354	Atrial Remodeling Following Catheter Ablation for Atrial Fibrillation-Mediated Cardiomyopathy: Long-Term Follow-Up of CAMERA-MRI Study.	CAMERA-MRI substudy; most patients. Crossed-over	https://dx.doi.org/10.1016/j.jacep.2019.03.009
31152874	Ablation versus Anti-Arrhythmic Therapy for Reducing All Hospital Episodes from Recurrent Atrial Fibrillation (AVATAR-AF): Design and rationale.	AVATAR Protocol	https://dx.doi.org/10.1016/j.ahj.2019.04.015
	Randomised Evaluation of the Impact of Catheter Ablation on Cognitive Function in Atrial Fibrillation.	REMEDIAL trial sub-study	https://dx.doi.org/10.1016/j.hlc.2022.06.187
	Catheter Ablation in Atrial Fibrillation and Heart Failure With Preserved Ejection Fraction Improves Peak Pulmonary Capillary Wedge Pressure, Exercise Capacity and Quality of Life. A Prospective Randomised Controlled Trial (RCT-STALL HFpEF).	RCT-STALL HFpEF preliminary results	https://dx.doi.org/10.1016/j.hlc.2022.06.021

Supplementary Table 3. Ongoing Trials

Registration ID	Year	Title	Source
jRCT1052210013	2021	Periprocedural Cerebral Embolism by Catheter Ablation of Atrial Fibrillation	https://trialsearch.who.int/Trial2.aspx?TrialID=JPRN-jRCT1052210013
jRCT1052200120	2021	Catheter Ablation in Asymptomatic Patients with Atrial Fibrillation	https://trialsearch.who.int/Trial2.aspx?TrialID=JPRN-jRCT1052200120
jRCTs031180249	2019	STroke secondary prevention with catheter ABLation and EDoxaban for patients with non-valvular atrial fibrillation	https://trialsearch.who.int/Trial2.aspx?TrialID=JPRN-jRCTs031180249
ACTRN12620000502932	2020	CAMERA-MRI II trial: catheter Ablation versus Medical Rate Control of Atrial Fibrillation with Systolic Heart Failure and Myocardial Fibrosis - an MRI Guided Multi-Centre Randomised Controlled Clinical Trial	https://trialsearch.who.int/Trial2.aspx?TrialID=ACTRN12620000502932
jRCTs042200051	2020	Catheter Ablation for Atrial Fibrillation After Percutaneous Mitral Valve Repair	https://trialsearch.who.int/Trial2.aspx?TrialID=JPRN-jRCTs042200051
NCT04272762	2020	Catheter Ablation in Symptomatic Atrial Fibrillation	https://clinicaltrials.gov/show/NCT04272762
KCT0008136	2023	EMOTIon and COgNitive function after atrial fibrillation catheter ablation vs. medical therapy; randomized clinical trial (EMOTICON trial)	https://trialsearch.who.int/Trial2.aspx?TrialID=KCT0008136
NCT05119231	2021	Pulmonary Vein Isolation vs SHAM-pulmonary Vein Isolation for Symptomatic Relief in Patients With AF- a Randomised Trial	https://clinicaltrials.gov/show/NCT05119231
NCT04037397	2019	First Line Radiofrequency Ablation Versus Antiarrhythmic Drugs for Persistent Atrial Fibrillation Treatment (RAAFT-3)	https://clinicaltrials.gov/show/NCT04037397
NCT05294445	2022	From the Emergency Department Directly to Ablation of Atrial Fibrillation Study	https://clinicaltrials.gov/show/NCT05294445
NCT04282850	2020	Ablation Versus Medical Management of Atrial Fibrillation in HFpEF	https://clinicaltrials.gov/show/NCT04282850
NCT04160000	2019	Treatment Of Atrial Fibrillation In Preserved Cardiac Function Heart Failure	https://clinicaltrials.gov/show/NCT04160000
NCT05514860	2022	The Impact of "First-Line" Rhythm Therapy on AF Progression	https://clinicaltrials.gov/show/NCT05514860
ISRCTN38385178	2018	A randomised comparison of cardioversion and catheter cryoablation in patients with persistent atrial fibrillation	https://trialsearch.who.int/Trial2.aspx?TrialID=ISRCTN38385178
NCT06125925	2023	Catheter Ablation in Atrial Fibrillation Patients With HFpEF (STABLE-SR IV Trial)	https://clinicaltrials.gov/ct2/show/NCT06125925
NCT05827172	2023	AF Ablation for HF With Reduced EF	https://clinicaltrials.gov/show/NCT05827172
NCT04011800	2019	Catheter Ablation vs. Risk Factor Modification (PRAGUE-25 Trial)	https://clinicaltrials.gov/show/NCT04011800<br>PMID: 35705334
NCT05717725	2023	Pulsed-field Ablation vs. Sham Ablation to Treat AF	https://clinicaltrials.gov/show/NCT05717725
NCT05508256	2022	CAtheter-Based Ablation of Atrial Fibrillation vs. Conventional Treatment in Patients With Heart Failure With Preserved Ejection Fraction	https://clinicaltrials.gov/show/NCT05508256
NCT05939076	2023	First-line Cryoablation for Early Treatment of Persistent Atrial Fibrillation	https://clinicaltrials.gov/ct2/show/NCT05939076
	2020	A randomised sham-controlled study of pulmonary vein isolation in symptomatic atrial fibrillation (The SHAM-PVI study)	https://doi.org/10.1002/clc.24066

Supplementary Table 4. Information on procedure and management of controls and complications

Study, enrolment	Ablation procedure	Catheters	Medical Therapy at Baseline	% AF ablation in controls (cross-over)	Complications
CAMTAF 2014<br><br>06/2005 to 07/2011	3D Mapping 100%<br>WACA 100%<br>1.7±0.7 Abl per pt	Irrigated RF	BB, ACEi/ARB-II, & spironolactone if LVEF<35% and NYHA≥3	0%	Abl<br>-Tamponade 4% (1); Stroke 4% (1)
RAAFT-AF 2 2014<br><br>07/2006 to 01/2010	3D Mapping 86%<br>CPVI 87%<br>Additional lesions 17-21%<br>1.2±0.4 Abl per pt	Irrigated RF	69% flecainide<br>25% propafenone<br>3% dronedarone<br>16.4% >1 anti-arrhythmic drug	48% (29)	Abl<br>-Tamponade 6% (4); PV stenosis 2% (1); Bradycardia needing pacing 2% (1)
AATAC 2016<br><br>10/2008 to 12/2012	3D Mapping 100%<br>PV antrum isolation 100%<br>Posterior Box 80%<br>SVC ± CFAE & non-PV triggers<br>1.4±0.6 Abl per pt	Irrigated RF	Amiodarone 100%<br>BB 80%, ACEi/ARB-II 88%, diuretics, digoxin; spironolactone 50% (NYHA=3)	NA	Abl<br>-Hematoma 2% (2); Pericardial effusion 1% (1)<br>Med/Amiodarone:<br>Thyroid tox 4% (4); Pulm tox 2% (2); Liver dysf 1% (1)
Sohara et al. 2016<br><br>2012 to 2013	Fluoroscopy<br>Thermal energy 26 to 33mm balloon<br>PVI 98.0%<br>Single Abl procedure	SATAKE Hotballoon	NA<br>28-day drug adjustment period to determine which AAD to be used	79% (34)	Abl+Crossover 134pt<br>-Stroke 1% (2); Complete AV block 1% (1); PV Stenosis 5% (7); Phrenic nerve palsy 4% (5)
CAMERA-MRI 2017<br><br>09/2013 to 12/2016	3D Mapping 100%<br>WACA – CPVI 100%<br>Roof and inferior lines (100%) – PWI attempted in 94% and achieved in 85%<br>1.4±0.6 Abl per pt	Irrigated RF	ACEi/ARB-II 94%, BB 94%, spironolactone 48%, AADs 24%	9.1% (3)	Abl<br>-femoral bleeding requiring transfusion 3% (1); Pneumonia 3% (1)<br>Med<br>-unplanned admissions 12.1% (4) [due to acute HF or need of ICD insertion]
CASTLE-AF 2018<br><br>01/2008 to 01/2016	3D Mapping 100%<br>Circular PVI 98.7%<br>Additional lesions 51.7%<br>1.3±0.5 Abl per pt	RF	ACEi/ARB-II 90%, BB 94%, diuretics (including spironolactone) 91%, digoxin 30%, AADs 27% (amiodarone 24%)	9.8% (18)	Abl<br>-pericardial effusion 1.7% (3); pericardial effusion requiring pericardiocentesis 0.6% (1); femoral bleeding requiring transfusion 1.7% (3; including 1 pseudoaneurysm requiring surgery); asymptomatic PV stenosis 0.6% (1); pneumonia 1.7% (3); groin infection 0.6% (1); worsening HF 0.6% (1); fever 0.6% (1); minor bleeding 1.2% (2)
AMICA 2019<br><br>01/2008 to 06/2016	3D Mapping 100%<br>WACA 100%<br>Circular PVI 97%<br>Linear lesions 33%<br>CFAEs 10%<br>1.2±0.5 Abl per pt	RF	ACEi/ARB-II 94%, BB 93%, diuretics 83%, aldosterone antagonists 67%, amiodarone 38%, digoxin 29%	0%	Abl - complications in 8.8% (6):<br>-atrioesophageal fistula leading to death (1); cardiogenic shock (1); pericardial tamponade (1); pleural effusion (1); suspected pericarditis (1); damage of ICD system (1); vascular access (2)
CABANA 2019<br><br>11/2009 to 4/2016	PVI<br>Additional lesions at operator discretion<br>Repeat Abl procedure in 19.4%	N/A	BB 67%<br>Calcium blocker 28%<br>Digoxin 9%<br>AADs 37%	27.5% (301)	Abl<br>-Femoral access 3.9% (39); Tamponade 0.8% (8); TIA 0.3% (3); MI 0.1% (1); Phrenic nerve injury 0.1% (1); PV stenosis >75%; Esophageal ulcer 0.5% (5); Severe pericardial chest pain 1.1% (11)<br>Med<br>-VT/VF 0.8%; Hypo- or hyperthyroidism 1.8%; Pulmonary toxicity 0.1%; Liver injury/failure 0.3%; Hypotension 0.3%; Allergic reaction 0.6%

CAPAPAF 2019<br>07/2016 to 2017	LA ablation for persistent AF<br>Number of repeat Abl procedures: N/A	N/A	Cardioversion in 100% or controls	N/A	N/A
CAPTAF 2019<br>07/2008 to 05/2013	Circular PVI in 93.3%<br>Roof line in 1.3%<br>Repeat Abl procedure in 18.7%	Irrigated-tip catheter 80%<br>Cryoballoon 20%	BB only 51.3% (39)<br>Class I or III AADs 44.7% (34)	10.8% (8)	Abl<br>-Access site 3.8% (3); Tamponade 2.5% (2); TIA 1.3% (1); UTI/Urosepsis 5.1% (4)<br>Med<br>-VT 2.6% (2); Bradycardia/AV block II/sinus arrest 2.6% (2); Non arrhythmic drug-related 6.6% (5)
ATTEST 2020<br>02/2012 to 02/2018	Circular PVI 100%<br>Repeat Abl procedure in 17.1%	Irrigated-tip catheter, with or without CF sensing	BB 61.4%<br>Class I or III AADs 54.3% (69)<br>[at baseline]	11.8% (15)	Abl<br>-Access site 2.0% (2); Pericardial effusion 1% (1); Tamponade 1% (1); Intracardiac thrombus 1% (1); Pericarditis 2% (2); Congestive heart failure 1% (1)
STOP AF First 2020<br>06/2017 to 05/2019	Circular PVI 98.1%<br>Single Abl procedure	Cryoballoon	BB 9%<br>Calcium channel blocker 4%<br>Class I or III AADs 89.4% [end of blanking period]	15% (15)	Abl<br>-Clinically significant pericardial effusion 1% (1); Myocardial infarction 1% (1); Ventricular tachyarrhythmia 1% (1)
EARLY-AF 2021<br>01/2017 to 12/2018	Fluoroscopy<br>PVI 100%<br>Repeat Abl procedure in 17.5%	Cryoballoon catheter (Arctic Front / Medtronic)	Beta-blocker 61.7%, CCB 6.7%, ACEi or ARB 26.2%	0%	Abl<br>- phrenic nerve palsy (3);<br>Med<br>-wide-complex-tachycardia (2); 1 syncope; 1 exacerbation of HF; Symptomatic bradycardia with pacemaker needed
CAPA 2021<br>03/2012 to 06/2014	3D Mapping 100%<br>Stepwise strategy: (i) complete PVI 96.5% (ii) Linear ablation if SR was not achieved in 42.8%; (iii) Ablation of the left atrial complex fractionated atrial electrograms in 5.2%<br>Repeat Abl procedure in 45.0%	Irrigated RF	Amiodarone 68.8%, Propafenone 73.6%.	0%	Abl<br>-Tamponade 2.4%; Haematoma 1.8%; Femoral arteriovenous fistula 0.9%; Stroke 0.3%; PV stenosis 0.3%; Gastroplegia 0.3%; Haemoptysis 0.3%<br>Med<br>-Thyroid dysfunction 5.3%; Symptomatic bradycardia 8.7%; Torsade de pointes 0.3%
Cryo-FIRST 2021<br>04/14 to 10/2018	Fluoroscopy<br>PVI 100%<br>Repeat Abl procedure in 5.6%	Arctic Front Advance Cardiac Cryoablation Catheter,	BB 50.5%, CCB 11.0%<br>60.4% flecainide, 32.7% propafenone, 5.0% sotalol & 1.9% dronedarone	17.1% (19)	Abl<br>-Arteriospasm coronary (1); Pneumonia (1); impaired gastric emptying (1); TIA (1); vascular access bleeding (1); pyrexia (1); pericardial disorders without need of pericardiocentesis (3)<br>Med<br>-adverse drug reactions (2)
RAFT-AF 2022<br>12/2011 to 01/2018	PVI 100%; persistent AF pts underwent additional ablation including ablation of complex fractionated atrial electrograms, roof line, mitral isthmus line, left atrial posterior wall isolation, or combinations.<br>Repeat Abl procedure in 37.6%	Irrigated RF	Antiarrhythmic medication 39.1%, BB 92.4%, CCB 23.4%, Digoxin 33%	0%	Abl<br>-death due to atrioesophageal fistula (1); effusions requiring pericardiocentesis (6); major bleeding events (8); minor bleeding events (5)<br>Med<br>-Significant bradycardia (4); Amiodarone toxicity (1)
AVATAR 2022<br>04/2015 to 08/2017	Randomization 1:1:1<br>PVI without confirmation in one group, confirmed PVI in another group with circular mapping catheter<br>Repeat Abl procedure in 12.0%	Arctic Front Advance™ cryoballoon	Class I/III AA 33%, BB 52.4%, CCB 6.8%.	0%	Avatar protocol:<br>-vascular complications (3); cardiac tamponade (2)<br>Conventional ablation arm:<br>-vascular complication (1); TIA (1)<br>Antiarrhythmic drug arm:<br>-death (1; sotalol); TIA (1); drug side effects/allergic reactions (18)
Ding et al. 2022<br>01/2018 to 11/2018	PVI in 100%<br>Single Abl procedure	Arctic Front Advance™ cryoballoon	100% on class I or III AAD	5% (5)	Abl<br>-Vascular complications (3); pericardial effusion (1); phrenic nerve palsy (2); impaired gastric emptying (1); bradycardia (2); tachycardia (3); bleeding events (1)

					Med<br>-bradycardia (4); tachycardia (1); bleeding events (3); adverse drug reaction (3)
CASTLE-HTx 2023<br>11/2020 to 05/2022	3D mapping 100%.<br>PVI 100%<br>Additional lesions 37%<br>Repeat Abl procedure in 9.3%	Irrigated RF	Amiodarone 46%, BB 91%, acei or arb 41%, SGLT-2 inhibitor, 25%, sacubitril/valsartan 57%	16%	Ablation<br>-vascular access complications (3)
RCT-STALL HFpEF 2023<br>12/2018 to 08/2021	3D Mapping 100%<br>PVI 100%<br>PWI for persistent AF 81.3%<br>Single Abl procedure	Irrigated RF	Amiodarone 6.7%, Sotalol 33.3%, BB 33.3%, Flecainide 26.7%, anticoagulation 93.3%.	0%	No complications reported.
REMEDIAL 2023<br>06/2018 to 03/2021	3D mapping 100%<br>PVI 100%<br>Additional ablation in 20%: PWI & CTI<br>Single Abl procedure	Irrigated RF contact-force sensing: SmartTouch ST or Tacticath	Amiodarone 13% (6), Flecainide 26% (12) and Sotalol 51% (24)	6% (3)	No major complications
ORBITA-AF feasibility 2024<br>10/2021 to 04/2022	Fluoroscopy<br>PVI 100%<br>Repeat Abl procedure in 30.0%	Medtronic Arctic FrontTM Advance	Sham-procedure / Cardioversion<br>AADs at baseline 60% (6) but stopped within 6 weeks to 3 months.	0% (0)	Abl<br>- bleed from ILR site (1); temporary phrenic nerve palsy (1)<br>Med<br>- chest pain (2); decompensated HF (1)

Supplementary Table 5. Information on Follow-up, AF relapse and Primary Endpoint definition

	Follow-up duration	Rhythm Monitoring	Definition of Relapse	Primary Endpoint Definition	Comments
CAMTAF 2014	6M both groups<br>12M Abl	Clinical, ECG, 48h Holter at 1, 3 & 6 months	AF/atrial tachycardia lasting ≥ 30s seconds after the 3M blanking period	Difference between groups in LV ejection fraction at 6 months determined by transthoracic echocardiography.	-
RAAFT-AF 2 2014	2 years	Clinical at<br>1, 3,6, 12 & 24M<br>Transtelephonic monitoring system – if symptoms & biweekly asymptomatic recordings	AF/ atrial tachyarrhythmias lasting >30s after the 3M blanking period	Time to the first documented atrial tachyarrhythmia of >30s (symptomatic or asymptomatic AF, atrial flutter, or atrial tachycardia), detected by either scheduled or unscheduled ECG, Holter, transtelephonic monitor, or rhythm strip.	2 Abl pt did not receive ablation
AATAC 2016	2 years	Dual-chamber ICD<br>or CRT-D	AF/ atrial flutter or atrial tachyarrhythmias lasting >30s off antiarrhythmics after the 3M blanking period	Long-term procedural success: freedom from AF, atrial flutter, or atrial tachycardia of >30 seconds duration off AADs at follow-up.	-
Sohara et al. 2016	12M	Clinical at 1, 3, 6 & 12M, weekly ECG, portable ECG monitor	AF lasting >30s after the 84-day blanking period	Chronic success: no documented AF episode continuing for ≥30s, regardless of the presence or absence of symptoms, and no use of restricted concomitant drugs or therapies.	As treated analysis:<br>4 pt in the Abl group and 6 pt in the Med group were excluded or withdrew
CAMERA-MRI 2017	6 months	ILR	AF/atrial tachycardia lasting ≥ 30s after the 3M blanking period	Change in LVEF from baseline at 6 months on cardiac MRI.	1 patient (3%) in the ablation arm did not receive ablation
CASTLE-AF 2018	Ablation: 37.6±20.4 months<br>Medical: 37.4±17.7 months	ICD<br>or CRT-D	Any atrial arrhythmia lasting >30s after the 3M blanking period	Composite of death from any cause or hospitalization for worsening heart failure.	28 patients (15.6%) in the ablation arm did not receive ablation (crossed over to medical therapy)
AMICA 2019	12 months	Daily ECG via external non-invasive device, 12-lead ECG during FU visits at 1, 3, 6 and 12 months<br>ICD/CRT-D in 75 pts (53.5%)	N/A (AF burden)	Absolute increase in LVEF from baseline at 1 year measured using transthoracic echocardiography.	1 patient (1.5%) in the ablation arm did not receive ablation
CABANA 2019	48.5 months (median)	ECG event recorder for 24-hour every 3 months + 96-hour ECG Holter every 6 months	AF lasting for > 30 sec after the 3M blanking period	Composite of death, disabling stroke, serious bleeding, or cardiac arrest.	102 patients (9.2%) in the ablation arm did not receive ablation
CAPAPAF 2019	6 months	ILR in all patients	N/A	Time to recurrence of persistent AF within 12 months	Only sleep sub-study was published.<br>Has 2nd control group with AV node Abl + pacemaker implantation
CAPTAF 2019	12 months	ILR or PPM + 12-lead ECG every 3 months and 24-hour ECG Holter every 6 months	AF burden	Quality of life: General Health subscale score (Medical Outcomes Study 36-Item Short-Form Health Survey) at baseline and 12 months.	4 patients (5.1%) in the ablation arm did not receive ablation
ATTEST 2020	36 months	Transtelephonic monitoring > 30s, weekly from month 3 to 9 and monthly thereafter	AF episode lasting > 7 days or requiring cardioversion after 48 hours after the 3M blanking period	Progression from paroxysmal to persistent AF: rate of persistent AF/atrial tachycardia at 3 years	26 patients (11.4%) in the ablation arm did not receive ablation
STOP AF First 2020	12 months	- 12-lead ECG at 1, 3, 6 and 12 months	AF/AT > 30 seconds during 24-hour ECG ambulatory monitoring or > 10 seconds	Treatment success at 12 months / Composite of: freedom from initial failure of the procedure; any subsequent AF surgery or ablation in the LA (including	-

		- 24-hour ECG Holter at 6 and 12 months<br>- Patient-activated telephone monitoring (weekly and when symptoms developed)	on 12-lead ECG after the 3M blanking period	those performed during the blanking period); or atrial arrhythmia recurrence (documented AF, atrial tachycardia, or atrial flutter for ≥30s during ambulatory monitoring or for ≥10 seconds on a 12-lead ECG), cardioversion, or use of class I or III antiarrhythmic drugs (ablation group only) outside the 90-day blanking period.	
EARLY-AF 2021	12M	Telephone call at 7 days and visits at 3, 6, and 12 months. Automatic transmissions from the implantable cardiac monitor on a daily basis, and manual transmissions at least weekly. Patient-controlled handheld telemetry device for symptomatic arrhythmia.	AF/Atrial flutter or atrial tachyarrhythmias documented by 12lead ECG, surface ECG rhythm strips, ambulatory ECG monitor, or on implantable loop recorder and lasting 120 seconds or longer.	First documented recurrence of any atrial tachyarrhythmia (AF, atrial flutter, or atrial tachycardia) between 91 and 365 days after catheter ablation or the initiation of an antiarrhythmic drug.	
CAPA 2021	Mean 54.2 ± 10.6M	Clinic visit after the initial therapy (both ablation and drug therapy), and subsequently with ECG, echocardiogram, Holter monitoring and 1-week event recorder or 7-day Holter at 3, 6, 12, 18, 24, 30, 36, 42, 48, 54, and 60 months.	AF/ atrial flutter or atrial tachyarrhythmias of at least 30 s in duration, as documented by ECG or a device-recording system following the post-ablation blank period.	Four primary endpoints: stroke/TIA, systemic embolism, major bleeding, and new-onset congestive HF.	
Cryo-FIRST 2021	12M	Clinic with 12-lead ECG and 7-day Holter at 1, 3, 6, 9, and 12 months.	AF/ atrial flutter or atrial tachyarrhythmias lasting >30 s at 12 months documented by 7-day Holter ECG or any other ECG recording outside of the 90-day blanking period. Cardioversion and repeat CA for AA recurrence outside of the 90-day blanking period were also considered.	Freedom from any atrial arrhythmia recurrence (at least one episode of AF, atrial flutter, or atrial tachycardia) lasting >30s at 12months documented by 7-day Holter ECG or any other ECG recording outside of the 90-day blanking period.	
RAFT-AF 2022	Median 37.4M	Clinic with 12-lead ECG, 6-minute walk test and quality-of-life assessment at 2, 4, and 6 months, and then every 6 months for a minimum of 2 years, or until the end of follow-up.	AF relapse was not an endpoint. Presence of AF on 12-lead ECG was reported.	Composite of all-cause mortality and all HF events, with a minimum follow-up of 2 years.	
AVATAR 2022	12M	No monitoring. The endpoint was 'hospital episode', including inpatient stay, attendance to an emergency room, or outpatient clinic to be reviewed by an arrhythmia specialist.	AF relapse was not an endpoint.	Composite of all-cause mortality and HF events defined as an admission to a health care facility for >24 hours or clinically significant worsening HF leading to the administration of intravenous diuretic in an emergency department or unscheduled visit to a health care provider, and an increase in chronic HF therapy.	AF episodes which did not lead to "hospital episode" were not considered (only events requiring healthcare resources).
Ding et al. 2022	36M	Whenever subjects experienced arrhythmic symptoms for >1 day, they returned to the hospital for follow-up. Once atrial	AF relapse was not an endpoint	First occurrence of persistent atrial tachyarrhythmia (AF, atrial tachycardia, or atrial flutter lasting for >7 days), following a 90-day blanking period following Abl or AADs.	2 pts in the Abl group did not receive Abl.

		tachyarrhythmia was identified, 7-day Holter monitoring was initiated for 7 consecutive days.			
CASTLE-HTx 2023	554 days in ablation group, 534 days in medical therapy group	Follow-up at 3, 6, 12, 24 month and every 12 months afterwards. Home monitoring in patients with ICD/CRT device or implantable cardiac monitor.	Any sustained episode of AF lasting 30 s or more.<br><br>To estimate AF burden all episodes were considered regardless of duration.	Composite of death from any cause, implantation of a left ventricular assist device, or urgent heart transplantation.	
RCT-STALL HFpEF 2023	6M	Clinical at 3 and 6 months. Arrhythmia assessment using pre-existing implantable cardiac electronic devices (ICEDs), implantable loop recorders (ILRs), or twice daily electrocardiogram (ECG) monitoring with the KardiaMobile (AliveCor). If none of these options was feasible, 24-hour Holter monitoring at 3 and 6 months	Any atrial arrhythmia >30 seconds after a single ablation procedure on/off AADs.	Difference in peak pulmonary capillary wedge pressure on exercise right heart catheterization from baseline to 6 months.	
REMEDIAL 2023	12M	Continuous rhythm monitoring, using an ILR (Reveal LINQ, Medtronic) or a pre-existing dual-chamber device, or via twice daily monitoring with the KardiaMobile (AliveCor, USA) electrocardiography monitoring device. If none of these options was feasible, 24-hour Holter monitoring was performed at 3, 6, and 12 months during follow-up.	Documented AF or atrial tachycardia more than 30 seconds' duration following the 90-day blanking period.	1. Hospital emotional distress as measured using Hospital Anxiety and Depression Scale score.<br>2. Composite outcome of Cognitive ability as assessed by results of Trail Making tests A and B'.<br>3. Arrhythmia free survival at 12 months post catheter ablation.	2 pts in the ablation arm did not receive ablation
ORBITA-AF feasibility 2024	12M	ILR in all patients<br>Clinical assessment at 6 weeks, 3 and 12 months.	Recurrence of AF (episodes >30s) outside the blanking period of 6 weeks post index procedure.	Evaluate the maintenance of patient blinding (as measured by the blinding index).	Only 10% of total trial inclusion

Supplementary Table 6. Risk of Bias Assessment

	Random sequence generation (selection bias)	Allocation concealment (selection bias)	Blinding of participants and personnel (performance bias): All-Cause Mortality, Stroke & AF Burden	Blinding of participants and personnel (performance bias): All Other Outcomes	Blinding of outcome assessment (detection bias): All-Cause Mortality, Stroke & AF Burden	Blinding of outcome assessment (detection bias): All Other Outcomes	Incomplete outcome data (attrition bias)	Selective reporting (reporting bias)	Other bias
AATAC 2016	+	?	+	−	+	?	+	+	+
AMICA 2019	+	+	+	−	+	+	+	+	+
ATTEST 2020	?	?	+	−	+	?	+	?	+
AVATAR 2022	+	?	+	−	+	?	+	+	+
CABANA 2019	+	+	+	−	+	+	+	+	+
CAMERA-MRI 2017	+	?	+	−	+	+	+	+	+
CAMTAF 2014	+	+	+	−	+	?	+	+	+
CAPA 2021	?	?	+	−	+	?	+	?	−
CAPAPAF 2019	?	?	+	−	+	−	+	?	+
CAPTAF 2019	+	?	+	−	+	+	+	+	+
CASTLE-AF 2018	+	?	+	−	+	+	+	+	+
CASTLE-HTx 2023	+	?	+	−	+	?	+	+	+
Cryo-FIRST 2021	?	?	+	−	+	+	+	+	+
Ding 2022	?	?	+	−	+	−	−	?	−
EARLY-AF 2021	+	+	+	−	+	+	+	+	+
ORBITA-AF 2024	+	+	+	+	+	+	+	+	+
RAAFT-2 2014	+	?	+	−	+	+	+	?	+
RAFT-AF 2022	+	+	+	−	+	+	+	+	+
RCT-STALL HFpEF 2023	+	?	+	−	+	+	+	?	+
REMEDIAL 2023	?	+	+	−	+	+	+	+	+
Sohara 2016	?	?	+	−	+	?	+	?	−
STOP AF First 2020	?	?	+	−	+	+	+	+	+

Justification for decisions if provided on the online xls file

Annex A. Search Expression

a) Cochrane Central Register of Controlled Trials (CENTRAL)

#1 - "catheter ablation" AND "atrial fibrillation" AND ("trial")" with Publication Year from 2014 to 2024, in Trials
Issue 1 of 12, January 2024

➔ CENTRAL - 359 records removing duplicates

b) MEDLINE & Embase

1 ("catheter ablation" and "atrial fibrillation" and "trial").mp. [mp=ti, ab, hw, tn, ot, dm, mf, dv, kf, fx, dq, bt, nm, ox, px, rx, an, ui, sy, ux, mx]

2 limit 1 to human

3 limit 2 to humans

4 limit 3 to yr="2014 -Current"

5 limit 4 to "remove preprint records"

6 remove duplicates from 5

2774 records after removing duplicates

→ EMBASE – 1305 records; MEDLINE – 1469 records
